# The internationalization of renewable energy finance

**DOI:** 10.1016/j.isci.2025.112367

**Published:** 2025-04-06

**Authors:** Sara Eberhart, Tobias S. Schmidt, Bjarne Steffen, Florian Egli

**Affiliations:** 1 Energy and Technology Policy Group, Swiss Federal Institute of Technology, 8092 Zurich, Switzerland; 2 Institute of Science, Technology and Policy, Swiss Federal Institute of Technology, 8092 Zurich, Switzerland; 3 Climate Finance and Policy Group, Swiss Federal Institute of Technology, 8092 Zurich, Switzerland; 4 School of Social Sciences and Technology, Technical University of Munich, 80333 Munich, Germany

**Keywords:** Energy policy, Energy Resources

## Abstract

Mitigating climate change necessitates vast investments in clean energy technologies globally, requiring internationalization not only of knowledge, production, and policies but also finance. This article examines 42,291 renewable energy investment deals across OECD countries from 2004 to 2022, revealing highly international capital flows. Results show that $US 45.4B annually (45%) is invested across borders, with varying degrees between countries. Further, renewable energy investments increasingly mirror general foreign direct investment (89% correlation), indicating financial mainstreaming, which can contribute to rapid deployment. These results are primarily driven by onshore wind and solar photovoltaic (PV). While all technologies experienced increased international capital mobility, offshore wind leads with 76% of capital exiting borders, whereas biomass is largely financed domestically (36% international). In sum, while international capital mobility has been crucial for growth in RE deployment, substantial country and technology differences exist. Our findings offer insights for novel low-carbon technologies and inform future research.

## Highlights

• Renewable energy capital out- and inflows vary strongly between countries
• Internationalization of biomass, wind, and solar investments has increased over time
• Offshore wind investments are most international, biomass the least
• International onshore wind and solar PV investments increasingly mirror FDI over time

## Graphical abstract

**Figure:**
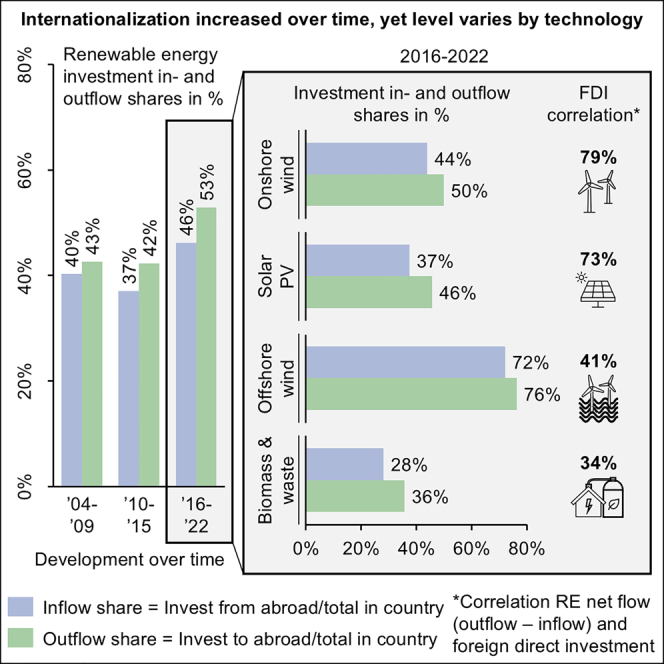

## Introduction

Limiting global warming to well below 2°C above pre-industrial levels necessitates a phase out of greenhouse gas emissions globally.^1^ Achieving this goal requires a global transition away from fossil fuel-based toward clean energy technologies.^2^^,^^3^ Importantly, clean energy technology innovation and manufacturing are geographically relatively concentrated,^4^^,^^5^ implying a critical role of cross-border flows to achieve an internationalization of the energy transition.^6^^,^^7^ Accordingly, a substantial body of literature has examined various dimensions of these cross-border dynamics, most prominently in the field of renewable energy (RE). This includes research on trade flows of materials and energy goods,^8^^,^^9^ the international flows of knowledge and learning,^10^ for example, in the context of knowledge spillovers,^11^^,^^12^^,^^13^ and the roles of global and local learning.^14^^,^^15^ Literature also extensively describes the internationalization of production with research into the significance of global supply chains for clean energy technologies.^16^ Further, the relevance of different actors in the transfer of technology and underlying knowledge across borders has been studied. This includes research on the role of technology suppliers^17^^,^^18^ as well as project developers.^19^ Another relevant aspect of internationalization in the energy transition is policies. Accordingly, both the effects and mechanisms of international climate change policies,^20^^,^^21^^,^^22^ as well as cross-border dynamics of national policies, meaning local policies spilling over to other jurisdictions^23^^,^^24^ have been researched.

A largely overlooked dimension of internationalization is that of clean energy asset finance, which, in contrast to other internationalization aspects of the energy transition, has received considerably less attention in the literature. Clean energy technologies are highly capital intensive; that is, a large share of their costs needs financing upfront.^25^^,^^26^ Investments into RE technologies alone need to reach at least $US 1.6 trillion p.a. by 2030.^27^^,^^28^ Importantly, capital flows can be much more dynamic than changing supply chains, where flexibility can often be challenging.^29^ Establishing new production facilities or relocating existing ones is generally a slower process than investing or divesting in renewable energy power plants for example, particularly as many of these projects are financed through project finance structures.^30^ When the aspect of cross-border financing for the energy transition has been explored, the focus has largely been on development finance and aid, particularly in the context of supporting clean energy deployment in emerging and developing economies.^31^^,^^32^^,^^33^^,^^34^^,^^35^^,^^36^ Yet – as we will show – the largest shares of cross-border investments do actually flow between OECD countries. Further, while there is some research on green foreign direct investment (i.e., not only real asset energy financing)^37^^,^^38^ as well as the internationalization of institutional (finance) investor holdings in low-carbon infrastructure real assets for 2020^39^ that also cover the OECD,^39^ we are lacking a comprehensive analysis of investment flow dynamics over time and by technology for such energy assets. Yet such insights would be needed to get a more complete understanding of the internationalization dynamics of the energy transition as a whole and provide implications for the global roll-out of less mature clean energy technologies.

Here, we thus compile detailed data of RE real asset investment flows across OECD countries from 2004 to 2022. We use three BloombergNEF^40^ (BNEF) datasets to calibrate random forest imputation models, resulting in a comprehensive dataset of 42,291 RE deals. We use this data to show differences in RE investment in- and outflows by country and compare this data to FDI to understand to what extent RE financing differs from general financial capital allocation, exposing technology differences.

We find that over time, the trajectory of RE real asset investments increasingly converged with the patterns of FDI for onshore wind and solar photovoltaic (PV), but this was less so for offshore wind and biomass. This is probably driven by mainstreaming effects through financial learning for such technologies. Further, the degree of internationalization has increased over time for all technologies, even though the level of internationalization differs by technologies. The most internationalized technology is offshore wind, while biomass had less cross-border investment flows. Onshore wind and solar PV fall in between the two extremes. Finally, in most cases, financial investors account for the largest shares of international investment, yet other investor types – including utilities – also invest abroad heavily. This is a step change for utilities as they usually have public mandates to provide energy locally, which did not require foreign investment in times of fossil fuel-based energy systems. Our findings thus provide an initial view of international clean energy cross-border financing dynamics based on the case of RE, where substantial historical data are already available as these technologies are relatively mature and widely deployed. We, therefore, discuss what the technology differences identified in the RE case imply for other clean energy technologies that have not yet reached a high level of (international) diffusion, such as long-term storage, electrolysers, or carbon capture and storage technologies (CCS). In addition, descriptive analyses serve as a valuable tool to help shape prospective research.^41^ Accordingly, we develop a future research agenda in the discussion.

## Results

### International renewable energy investment

**Figure 1:**
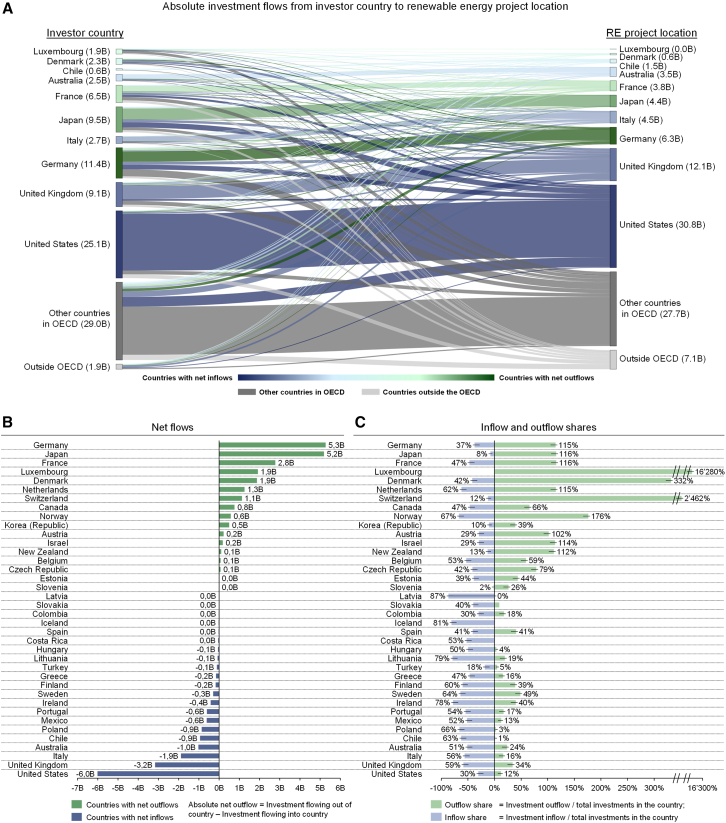
Annual RE investment flows per country 2004–2022 (A) Investment flows for all RE technologies for 10 countries with largest net in- and outflows averaged per year (see also Figure S1 for graph with all countries). (B) Net in- and outflows, averaged per year. (C) In- and outflow shares in relation to the total domestic RE investment sums (e.g., outflow share = investment outflow/total investments in the country) in the respective country. An investment flow is defined as an investment from a country where the equity or debt investor is located (outflow) to the country where the RE project is located (inflow). Data are aggregated across all RE technologies considered (onshore and offshore wind, solar photovoltaics (PV), solar thermal, biomass and waste, small-scale hydroelectric, and marine energy) from 2004 to 2022 and shown in 2020 US$.

In the first step of the analysis, we consider aggregated RE investment flows from 2004 to 2022 between OECD countries, as shown in Figure 1. We observe that RE investments differ substantially by country (Figure 1A), as do absolute and relative in- and outflows (Figures 1B and 1C). Germany and Japan stand out as key RE capital exporters with net investment outflows of US$ 5B or more on average per year. These investments predominantly went to the United States (US) and the United Kingdom (UK), which both are large RE capital importers with net inflows of $US 6B and $US 3B, respectively.

Naturally, larger economies tend to invest more in RE. To grasp the relative importance of cross-border flows by country, we divided inflows and outflows by the local investments in a country in Figure 1C. We see that for some of the countries that had large absolute flows, internationalization is actually less dominant – for example, in the case of the US, which had the largest net inflows but an inflow share of only 30%. By contrast, Germany and Japan not only prominently feature in the outflow category in absolute terms; they also remain among the larger outflow countries in terms of relative flows. Both are large export-oriented countries with an export-oriented financial system (including OEMs’ own financing branches, such as Siemens or Mitsubishi Financial Services). The highest relative outflows, however, are from Luxembourg, followed by Switzerland. The investment patterns of these countries are similar: substantial outflows, minimal inflows, and relatively modest domestic investment-making, resulting in extremely large outflow shares. Yet, the components for these patterns are distinctly different. The large sums of investments flowing out of Luxembourg are affected (85% of outflows) by the European Investment Bank (EIB) located in the country and investing all over Europe. Besides the EIB, there are banks, holding companies, and renewable energy enterprises that invest internationally out of Luxembourg. By contrast, Switzerland’s outflows to a large extent go to the US – affected by “Capital Dynamics”, a fund managing US pension money, registered in Switzerland. While this observation may raise the question if the identified patterns could be somewhat distorted due to this type of circularity of money – where investors may indirectly invest in their own countries via another, often through Offshore Financial Centers (OFCs) – we find no evidence to support this. Such cases represent a small minority in our study (see Table S1 for an analysis of US inflows), and recalculating net flows of Figure 1B, excluding OFCs (based on the IMF 2007 definition^35^) yields results consistent with our original analysis (see Figure S2). Other large investors from Switzerland include banks and also several utilities. This is a somewhat different picture than what we observe in Germany and Japan, where most of the largest international investors are banks – albeit in the case of Germany most of these banks are *Landesbanken* (i.e., public banks which have a history of investing in infrastructure), whereas in Japan the largest investors are private international financial investors (e.g., including Mitsubishi UFJ Financial Group).

### Renewable energy project finance versus foreign direct investment trends

We have shown that cross-border RE investment is common in OECD countries. Yet cross-border RE asset investments could face more obstacles than standard investments, impeding the international deployment of RE. To get a first understanding of whether such obstacles might indeed exist, we have a closer look at potential differences in net flow patterns of RE and standard investments by contrasting RE investments to FDI. Namely, we aggregate RE investment and FDI from 2004 to 2022 first and add a temporal perspective by splitting the 19 years of available data into an early (2004–2009), middle (2010–2015), and late period (2016–2022) second. While the first two periods encompass six years each, the last period was chosen to capture the years after the Paris Agreement and covers seven years. To allow comparisons between periods, investment numbers are annualized.

**Figure 2:**
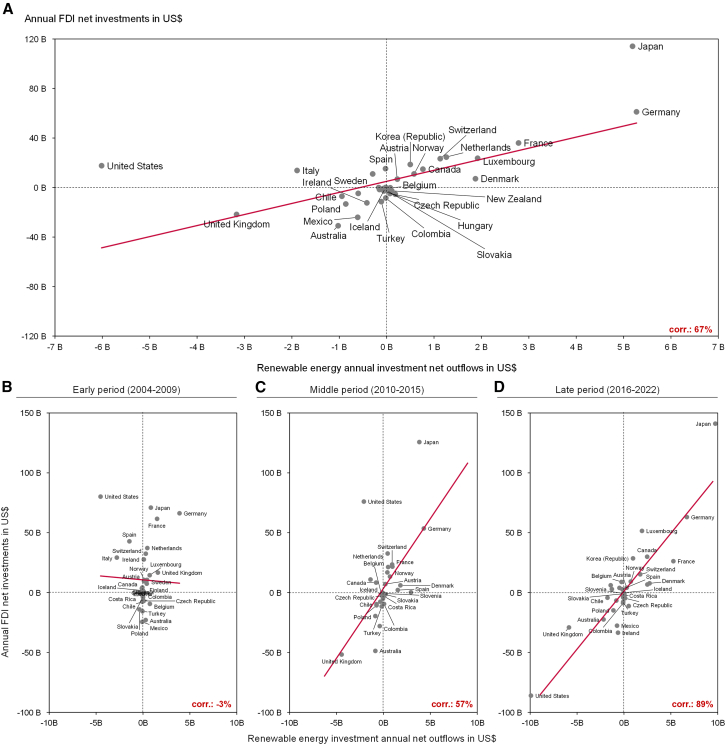
Absolute net investment flows into RE contrasted with foreign direct investments per country (A) Aggregated from 2004 to 2022. (B) Aggregated for the early period (2004–2009). (C) Aggregated for the middle period (2010–2015). (D) Aggregated for the late period after the Paris Agreement (2016–2022). The red line represents the trendline of the observations. “Corr.” Indicates the simple correlation between the net RE outflows and FDI. Data is aggregated across all RE technologies and is shown in 2020 US$.

Figure 2 shows that when aggregating the data over the entire 19 years, we observe a positive relationship between net FDIs and net RE outflows of countries (Figure 2A) and a correlation of 67%. This pattern suggests that the financing trends for RE projects follow similar patterns as those of financial investments. With Japan and the US, we observe two countries that appear as outliers to the trendline. Both have relatively higher net FDI than RE net outflows. In Japan, although RE investment outflows are substantial compared to other countries in our dataset, they do not match the magnitude of FDIs as observed in other countries. Conversely, in the US, while total net FDIs were positive, the country experienced large net inflows in terms of RE investments.

When dividing the data into different time periods in Figures 2B–2D, it becomes apparent that the positive overall correlation is the result of the periods starting in 2010. In the early period, some countries with substantial RE flows, such as the US, Spain, and Italy, experienced substantial inflows in RE investments despite having net positive FDI flows. This results in a correlation of −3% for that period. In the middle period, a positive trendline and correlation of 57% can already be observed. Again, the US and Japan are outliers. In the late period, an even clearer positive trend emerges, and larger previous outliers (such as the US) disappear, leading to a correlation of 89%.

These findings suggest a growing resemblance of RE investments and general financial flows – especially with growing RE investments and corresponding advances in financial learning for new technologies. So far, we have looked at RE as a homogeneous type of investment. However, technologies differ in terms of their need for customization, and thus the need for local learning and relatedly their technology-type-specific experience rates^42^ and financing conditions^43^ diverge. Accordingly, the patterns of internationalization in their financing might differ, too. In the following, we therefore move to a technology level.

### Technology differences

**Figure 3:**
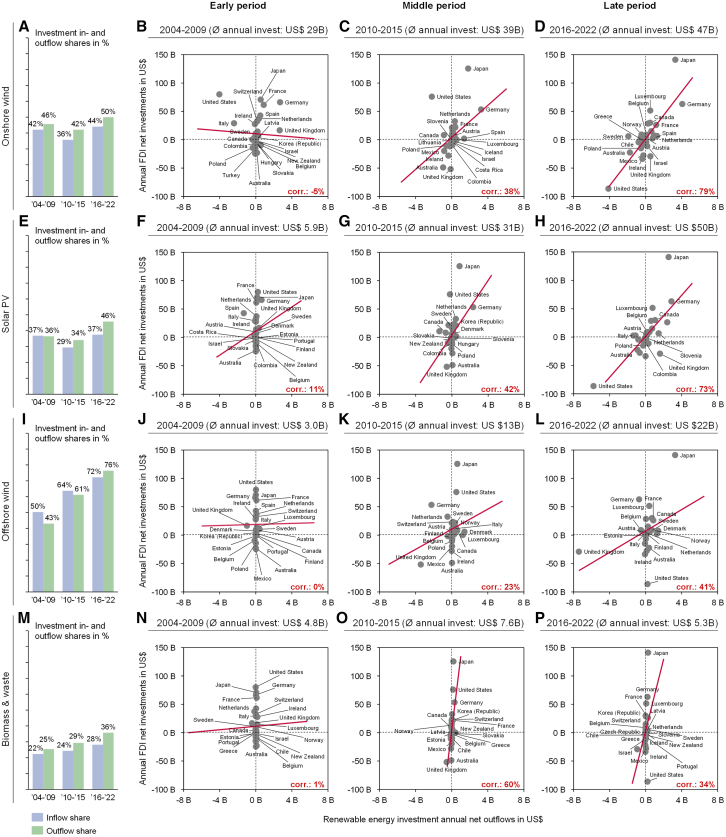
Internationalization by technology Investment inflow and outflow shares aggregated across all countries by technology: (A) onshore wind, (E) solar PV, (I) offshore wind, (M) biomass and waste. Absolute net investment flows into RE contrasted with foreign direct investments per country over three time periods by technology: (B–D) onshore wind, (F–H) solar PV, (J–L) offshore wind, (N–P) biomass and waste. The investment amounts found in the titles are total average annual investments across all OECD countries for the specific period and technology. The red line represents the trendline of the observations. “Corr.” Indicates the simple correlation between the net RE outflows and FDI. Absolute investment numbers are shown in 2020 US$. Note that investment flows are considered if they either originate from or are directed to an OECD country. Accordingly, some flows from and to non-OECD countries are included, and therefore, total aggregated in- and outflows (absolute and shares) are not equal.

To identify potential internationalization differences by technologies (e.g., due to their inherent characteristics), the previous analysis is conducted again, but disaggregated by the four largest RE technologies by total investment sums – covering 90% of investments – namely: onshore wind (Figures 3A–3D), solar PV (Figures 3E–3H), offshore wind (Figure 3I–3L), and biomass and waste (Figures 3M–3P).

A tendency of increasing inflow and outflow shares is visible across all technologies over time (Figures 3A, 3E, 3I, and 3M). However, the degree of internationalization differs substantially by technology. While in the most recent period, offshore wind investments have, for example, exhibited inflow and outflow shares of 72% and 76%, respectively (Figure 3I), such shares were at only 28% and 36% for biomass in the same period (Figure 3M). The increased internationalization in offshore wind projects may be due to their distinctly unique characteristics, including substantial investment needs (given large project sizes), project complexity, and duration, leading to higher investment risks (and thus financing costs).^44^^,^^45^ As a result, state investment banks (SIBs) often participate in offshore wind deals, for reasons including supporting their exporters.^46^ Further, offshore wind is often financed by large international energy providers (in some cases originating from the oil and gas industry), as well as by international conglomerates that also produce materials, parts, and other products.^45^ Given their business footprint, these investors are more likely to operate internationally. Conversely, the relatively lower internationalization levels for biomass could be connected to a number of different factors. For example, the localized nature of the resources used for this type of energy generation. Biomass technology requires substantial local adaptation and is thus more closely tied to local networks and supply chains.^19^ The observed localized financing of Biomass potentially also mirrors and could be connected to patterns in agricultural financing, which is typically locally embedded.^47^ Farmers traditionally have existing lender relationships and may thus rely on their usual banks for financing additional energy-related investments, such as biomass projects. Moreover, biomass projects differ substantially from other renewable technologies in their financing needs, as the requirement of feedstock makes them relatively OPEX-heavy.

Technologies also differ in terms of how closely their cross-border investment patterns resemble FDI flows over time. While no clear patterns or correlations can be observed for any of the technologies in the early period (Figures 3B, 3F, 3J, and 3N), this changes for the more recent periods. For onshore wind and solar PV, an alignment of energy investments with FDIs is evident for these periods (Figures 3D and 3H). Conversely, for offshore wind and biomass, little to no clear pattern can be observed in the same period (Figures 3L and 3P). A slight uptick in correlation in the medium term is apparent for biomass (Figure 3O). Hence, the patterns that were observed in the overall data were strongly driven by onshore wind and solar PV, which made up the majority of the investments (almost 80% in the latest period).

While we know that the classical electricity industry was more typically financed locally, we have now seen that there are large shares of internationalization in the financing of RE technologies. The question thus is: Who might be driving such cross-border flows? To answer that, a better understanding of the internationalization tendencies of different investor types is required.

### Differences in investor types

In the final step of our analysis, we examine to what extent different investor types drive internationalization by comparing their propensity to invest locally versus abroad. We examine equity investments for three reasons. First, our data indicates that equity investors include a broader range of types of investors compared to debt providers (predominantly banks), making it a more interesting focus for analysis. Second, equity investors play a critical role in determining the locations of RE projects, as they are the ones making strategic decisions about where to develop projects, whether to expand internationally, and how to approach business development.^19^ Consequently, this then may impact internationalization. Third, an equivalent analysis for debt investors is not feasible due to data limitations. Many debt-financed deals in the original dataset lacked detailed investor information, often labeled as "private" or "not reported," and had to be excluded (see method details for more details). Consequently, any analysis of debt investor types specifically carries the risk of yielding skewed results.

**Figure 4:**
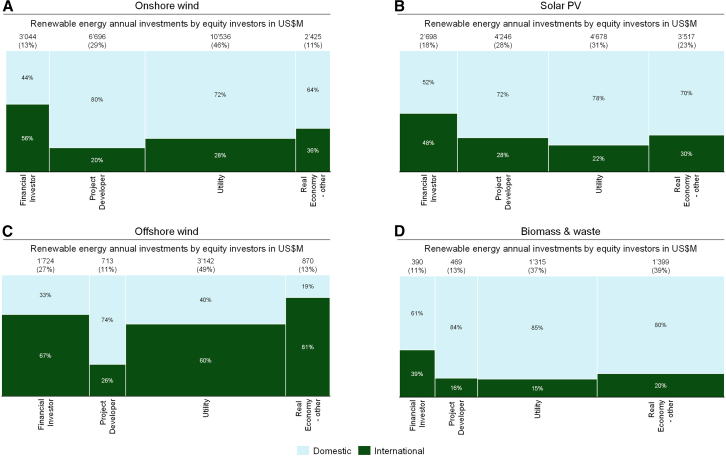
Domestic and international equity investments by investor types By technologies: (A) onshore wind, (B) solar PV, (C) offshore wind, and (D) biomass and waste. Data are considered from 2004 to 2022 and absolute investment numbers are shown in 2020 US$. The area of each chart reflects the relative distribution of domestic versus international investments and the relative share invested by type of equity investor. The shares of domestic and international investments per investor type are indicated by the percentage numbers within the areas. The absolute annualized investment amounts and the corresponding relative share of investments per investor type is indicated at the top of each column.

Figure 4 illustrates the relative relevance of different investor types in financing the technologies analyzed previously. It contrasts the shares of domestic and international investments (i.e., investments flowing outside the home market) over the entire time period. Of all the investor types analyzed, financial investors invest most internationally for all technologies except offshore wind. For that technology, investors that are neither financial investors nor fall into the project developer/utilities category show the largest shares of investment outflows. However, also in offshore wind, the share if international investments by financial investors is very high (67%, see Figure 4C). And at 27% of total investments, financial investors account for a relatively large share of total investments in offshore wind compared to other technologies. These findings support our assertion that large offshore wind internationalization shares might be partially driven by SIBs. Finally, upon examining the evolution of investor type internationalization shares over time (see Figure S3), we see that the importance of financial investors has progressively grown over time across all technologies except solar PV.

The lowest shares of internationalization are observed from project developers for both wind technologies. For solar PV and biomass, utilities have the lowest share of investment outflows. Yet, at 22% (Figure 4B) and 15% (Figure 4D), respectively, these are still noteworthy. At the same time, utilities are often the dominant investor type (except for biomass). Accordingly, the level of overall internationalization of a technology will also heavily depend on the level of internationalization of utility investors.

## Discussion

Our analysis deepens the understanding of RE financing flows across OECD countries, particularly by examining temporal trends and distinguishing between different technologies and investor types. We found that net investment flows for RE projects tend to start showing similar patterns as FDI flows over time, specifically for solar PV and onshore wind. Mainstreaming, fueled by financial learning, may be a driving factor as the financial market experiences learning over time and can better assess technologies and their risks.^43^ Investors gain confidence, and barriers to technology-specific project financing might become less relevant. This explanation is supported by the fact that especially onshore wind and solar PV are technologies that have reached a high degree of technological and financial maturity.^48^^,^^49^ Accordingly, such a correlation measure between FDIs and a technology’s internationalization patterns could potentially serve as an indicator to detect a trend toward (financial) maturity of future clean energy technologies. We further observed a consistent trend toward internationalization across all technologies, with offshore wind increasing most. In recent years, 76% of offshore wind investments from OECD countries went abroad, compared to only 36% in biomass.

Several of the findings from the case of RE asset finance internationalization can be used to derive initial high-level implications for future policy development both for RE and, importantly, also other, more nascent clean energy technologies. First, the prevalence and growth of internationalization and cross-border flows underscore that energy project financing relies heavily on international capital mobility and the markets governing such capital. Given the scale of deployment required to meet climate goals, this trend is likely to continue. We find that, particularly for large-scale projects, internationalization is important (see Figure S4 in the supplemental information). From an energy transition perspective, it is therefore crucial to minimize barriers obstructing international flows not only of technology^16^^,^^50^^,^^51^ but also of capital and thus promote financial collaboration between countries. Policymakers could, for example, standardize regulatory frameworks and foster bilateral or multilateral agreements to facilitate cross-border investment flows, thereby enhancing international cooperation. This is likely most important for technologies that require a high degree of regulatory intervention due to their inherent characteristics (such as safety, and so forth).^42^ Second, our results reveal varying investment patterns across countries. While general prescriptions for optimal in- or outflow patterns do not exist, such descriptive analyses can serve as indicators to flag countries with unusual flows that should be further examined to identify potential bottlenecks in investments. For instance, very low inflows could be seen as a potential flag to suggest further investigation. Such flows could either be explained by the fact that sufficient local capacity has been installed already or point toward a restrictive policy environment for clean energy deployment that might lead to insufficient inflows of financing. This might especially be the case when paired with a significant share of internationalization (i.e., capital outflow) among local utilities, which are typically expected to invest more locally. Such insights could then help countries to adjust their current energy and financial policy landscape. Third, our analysis shows that mainstreaming and internationalization patterns are specific to technologies. This could be an indicator that there are certain technology-inherent characteristics that will influence the technology’s financing in terms of internationalization – similar to local and global learning and international spillovers that have been found to be specific to a technology’s characteristics.^19^^,^^42^ Finally, the findings also align with previous evidence that finance is not technology neutral. Existing research suggests that both financing sources^52^ and structures^30^ vary for different technologies, and thus, their availability may shape, which technologies can be financed. Similarly, the observed differences in internationalization potential among technologies may carry comparable implications for less mature clean energy technologies. Of course, policymakers typically need to carefully balance many different goals, interests, and consequences when devising energy transition strategies and the policies to support them. Given the broader context of global capital constraints paired with substantial RE investment needs, prioritizing technologies that offer the highest marginal return and for which financing is most readily available might be one of these objectives. If devising such a goal in the context of our findings on technology differences in internationalization, it would mean that countries might want to factor in their ability to raise local capital as one of the criteria when shaping their energy strategy. For example, in a case where local capital is abundant, less internationalized technologies such as biomass (or in the future, bioenergy CCS) could be a more attractive investment option, and support for local developers and utilities might be prioritized. Conversely, in a country where local utilities are highly indebted, local capital is scarce, and public budgets are limited, technologies with more mainstreamed or internationalized capital flows – for example, wind energy (or, in the future, stationary lithium-ion battery storage) – might be a better strategy. In such cases, financial and energy policies could be structured in a way that the influx of foreign capital is incentivized, potentially through mechanisms such as tenders in hard currency, the facilitation of special purpose vehicles (SPVs), and other support mechanisms for project finance setups through de-risking policies.^30^ It might also be worth considering that a strategy based on higher levels of internationalization can help a country build resilience against instability in the local financing market. Finally, it should be noted that the implications discussed here are primarily relevant to OECD countries, as these were the focus of the study. Future research would be needed to extend the study to EMDEs (including China) to make global recommendations that include those regions.

These initial insights on key internationalization patterns in the financing of RE real asset projects further give direction on how the research could be further deepened for OECD countries. One direction might be an ongoing research program capturing evolving trends. Such research could offer tailored insights and recommendations for countries by examining drivers of cross-border flows and focusing on outlier countries to understand their investment patterns. For example, Japan’s role as an exceptionally important reference investor has been studied previously,^53^ however, why – in comparison to other branches of the economy – RE investments do not seem to reach the same level of magnitude in terms of outflows would need to be investigated further. Outside such a program, a promising avenue for future research lies in investigating how climate policy mixes and their design influences international investment flows in RE. This would involve exploring the mechanisms through which policy frameworks shape these flows and assessing their effectiveness across different contexts. For instance, the OECD policy database could serve as a valuable resource to identify and analyze such policy influences systematically. Additionally, as more data becomes available beyond 2022, it would be particularly interesting to examine the larger-scale impacts of major initiatives such as the EU Green Deal and the Inflation Reduction Act (IRA) in the United States, providing deeper insights into their role in driving global investment trends. In addition, and with an eye on the IRA and other recent developments that have been fueling geoeconomic fragmentation, another relevant avenue for future research could involve exploring how some of the observed internationalization trends might be reversed under increased fragmentation in different geopolitical scenarios. This could provide a deeper understanding of the vulnerabilities and resilience of international investment flows in RE amidst evolving global dynamics. More technology-focused future research could improve our understanding of the identified variances in internationalization patterns between technologies, for example, by a more formal analysis into the high internationalization of offshore wind or by identifying whether observed technology differences can be assigned to specific technology characteristics. Doing so would allow the research and its recommendations to be extended to more nascent technologies, for example, electrolysis or carbon capture and utilization (CCU). Finally, conducting further in-depth analyses on various investor types could provide a richer understanding of how these investors facilitate the movement of capital. For instance, the investment practices of utilities could be further investigated. Even though their inclination toward international investment seems to be limited in comparison to other investor types, they exhibit noteworthy levels of internationalization – despite their supposed focus on supplying energy domestically. Such additional insights on different investor types could help to inform policy and allow more targeted policies for activating different investors more purposefully.

### Limitations of the study

This study has three main limitations. First, our analysis of technology types is constrained by the database used. While we look at some of the largest renewable energy technologies—wind, solar, and biomass—we do not include large hydropower plants. Second, the database has limitations in terms of investment details, particularly regarding investment amounts, debt shares, and debt investor types because investment terms are usually not publicly disclosed. As a result, we had to drop some data (e.g., debt investments without investor information) and use data imputation (e.g., investment amounts and debt shares), as described in the method details. Given the missing data on debt investors, the investor type analysis is limited to equity investments. Further, data imputation introduces uncertainty on the data quality. We conducted a complete case analysis (i.e., ran the analysis only on the data where all the information is fully available) and Monte Carlo simulations as robustness checks. Both approaches confirmed that key insights remained unchanged (see method details). Finally, our analysis is limited to OECD countries, yet investment dynamics specific to developing economies are not captured, and thus the findings will not be directly applicable to those countries.

## Resource availability

### Lead contact

Inquiries regarding the data and method associated with this article can be directed to Sara Eberhart (sara.eberhart@gess.ethz.ch).

### Materials availability

This study did not generate new material.

### Data and code availability

• Data: The asset finance data used in this analysis is proprietary but can be accessed via a subscription model from BloombergNEF^40^ (BNEF). All other data are from public sources, specifically UNCTAD^54^ for FDI data and World Bank^55^ for US inflation data.
• Code: For replication purposes, the R code used for data preparation and imputation has been deposited on GitHub available at https://doi.org/10.5281/zenodo.15013912*.* Users are required to cite the code when using it for their research. Detailed licensing information is available within the repository.
• Other items: No other items were created or used in this study

## Acknowledgments

This work was supported by the 10.13039/501100005380Swiss Federal Office of Energy [contract number: SI/502527-01], but the authors bear the full responsibility for the content of the article as well as the conclusions and findings presented.

## Author contributions

Conceptualization, S.E., T.S., B.S., and F.E.; data curation, S.E.; formal analysis, S.E.; funding acquisition, F.E., T.S., and B.S.; investigation, S.E.; methodology, S.E., T.S., B.S., and F.E.; project administration, S.E.; software, S.E., supervision, T.S., B.S., and F.E.; visualization, S.E.; writing – original draft, S.E.; writing – review and editing, S.E., T.S., B.S., and F.E.

## Declaration of interests

The authors declare to have no competing interests.

## Declaration of generative AI and AI-assisted technologies in the writing process

During the preparation of this work the authors used ChatGPT in order to support language and readability editing. After using this tool/service, the authors reviewed and edited the content as needed and take full responsibility for the content of the publication.

## STAR★Methods

### Key resources table

**Table:** 

REAGENT or RESOURCE	SOURCE	IDENTIFIER
**Deposited data**		

Investment deal information	BNEF– Asset Finance^40^	https://about.bnef.com/
Asset/Project information	BNEF– Projects^40^	https://about.bnef.com/
Investor information	BNEF– Organizations^40^	https://about.bnef.com/
Foreign Direct Investments	UNCTAD^54^	https://unctad.org/data-visualization/global-foreign-direct-investment-flows-over-last-30-years
Inflation Data	World Bank^55^	https://data.worldbank.org/indicator/FP.CPI.TOTL.ZG?locations=US

**Software and algorithms**		

Excel	Microsoft	https://www.microsoft.com/en-us/microsoft-365/excel
R Software	Bell Laboratories	RRID:SCR_001905
Code for data preparation and imputation	This paper	https://doi.org/10.5281/zenodo.15013912

### Experimental model and study participant details

This study does not include experiments or subjects.

### Method details

#### Data sources

Our analysis is based on an investment dataset of 42,291 RE real asset deals with partially imputed information. This dataset is constructed from BloombergNEF^40^ (BNEF) data. BNEF is one of the most comprehensive sources for clean energy financing data, widely used by researchers and financial decisionmakers. For the purpose of our analysis, we require detailed technology information, location of the asset covered per deal (i.e. destination of financial flows), year of the deal, and investment values per investor per deal, as well as information on the investors, such as their industry and the country where that investor is located (source of financial flows). To acquire these information points, we combined the three BNEF datasets on asset finance transactions, projects, and organizations. Further, several data cleaning and imputation steps were necessary, as for some important variables, the dataset was missing datapoints (see below).

Besides the BNEF data, we used foreign direct investment (FDI) and inflation data. The FDI data was sourced from the United Nations Conference on Trade and Development (UNCTAD).^54^ The inflation data was sourced from the World Bank database.^55^ United States inflation rates were used to correct US$ values for inflation effects, using 2020 as the base year.

#### Scope of analysis

We focused our analysis on the four largest RE technologies by total deals/investments: onshore wind, solar PV, offshore wind, and biomass & waste. This covers 90% of the deals available in the BNEF asset finance dataset. For the analyses that are not technology specific, we also included a small number of “other” technologies, including solar thermal, small hydro, and marine, as well as wind and solar projects that could not be identified in further detail (see “data processing” for more information). BNEF excludes hydro power projects, whose potential for capacity additions in OECD countries is anyway limited.^56^

We considered deals with a date of close between 2004 and 2022: BNEF was established in 2004, and 2022 was the last full year available at the time of the analysis. To add a temporal perspective to our analysis, the data is split into three sub-periods, with an “early” period of 2004–2009), a “middle” period of 2010–2015, and a “late” period of 2016–2022, coinciding with the time after the Paris Agreement.

We focused our analysis on OECD countries. Accordingly, we only considered deals where either the project location was in an OECD country or the investor was located in an OECD country, or both.

Deals were included in the analysis when they were flagged as either “completed” or “announced/filed” (this excluded “abandoned”, “postponed/cancelled”, and “in active planning”). In addition, all transaction types of deals (i.e. “new build”, “acquisition”, and “refinancing”) were used for the analysis as our interest lies in overall capital flows into powerplants, not only newly developed projects – see Figure S5 for detailed information on the relative importance of the transaction types in the final data. Additionally, we have conducted all analyses again excluding acquisitions and refinancing, with the results presented in Figures S9–S14. As shown, all key findings hold – independent of the in- or exclusion of these transaction types.

#### Data processing

When preparing the “raw dataset”, several data processing steps were necessary. This included merging the three BNEF datasets, classifying investor types, and assigning total investment values to individual investors involved in a deal. This processing was done as follows:

Project country (used as the destination country in our analysis), project size in MW, deal values in US$, investor names, and year of close of RE asset deals were drawn from the asset finance dataset. Given a lack of detail with regards to the technology types (i.e. onshore vs. offshore wind and solar PV vs. concentrated solar thermal) within the asset finance dataset, we added this information from the BNEF’s project dataset by combining the two datasets via their project/asset finance IDs (mapping provided by BNEF). In some cases, several projects are mapped to a single deal, as certain transactions financed more than one asset/plant. For these instances (n = 334), we manually adjusted and controlled the data to split such deals into the respective projects according to the detailed information provided in the deal description fields. For example, if a deal covered the purchase of both solar and wind projects, the deal would be split into two separate deals/lines (one assigned to wind and one to solar in the technology field) and the MW and investment values assigned respectively. Deals that required this adjustment were either portfolio (i.e. several projects that differed in technology and were in different locations were financed with one transaction) or co-located deals (i.e. in one location, a project was financed that encompassed two or more different technologies). Where such an adjustment was made, the deals were flagged in the dataset as “portfolio” or “co-located”, respectively. In addition, a small share of deals could not be mapped to the project data at all, and thus a distinction within the wind and solar categories was not possible. Such deals (making up 5.4% of total MW in the final dataset) were assigned to the “others” category, which covers deals where the technology is unclear or that are for a technology that is not in the focus of our analysis (see “scope of analysis” for more detail).

From the third BNEF dataset (organizations), we used key investor information, such as the country where the investor company is incorporated (used as the investment source country in our analysis), as well as information to identify the investor’s industry. The investor industry was required to classify the organizations into distinct investor types. Given differences between investor types in relation to their risk/return expectations, knowledge bases, preferred financing structures and cost of capital, we follow previous research^30^^,^^57^ and split the investor types according to perceived differences in their motivation for investing in RE, namely utilities (provide energy as a public service to businesses and households), project developer (responsible for identifying, developing, and managing the construction of energy projects), and financial investors (financial institutions like banks, private equity funds, etc. that invest out of financial interest). Any company not matching any of these categories, was classified as a “real economy – other” investor. Note that in our analysis, investor types are only applicable for equity investors.

The investor industry was not a variable readily available in the BNEF “organizations” dataset. We therefore had to source it from an additional Bloomberg database or identify based on a “sub-activity” variable. Where Bloomberg Tickers (Bloomberg-specific identification numbers for companies or assets) were available in our BNEF dataset, we were able to directly source the industry information in the form of the “Bloomberg Industry Classification Standard” (BICS) from Bloomberg and then map these classifiers into the four defined investor types (see supplementary excel mapping file Table S5). For the remainder of the data – which amounted to around 40% of the companies in the organization dataset – we had to determine the investor class with the help of the “sub-activity” reported in the BNEF organizations data, where that field was available. A total of 716 sub-activities were each manually mapped (using data where both sub-activities and BICS classifiers were known as indicative support) to one of the four investor type classes (see supplementary excel mapping file Table S6). Organizations with one sub-activity only were assigned the respective investor type. In the case of several sub-activities, the investor was assigned according to the following rules: utilities, if one of the sub-activities was flagged as “utility”, otherwise project developers, if one of the sub-activities was flagged as “project developer”; otherwise, the investor class emerging most often. In the case that several investor types were listed the same number of times, the investor class corresponding to the sub-activity listed first in the data was used. In the final dataset used to analyse the equity investor types, 3.5% of total investments could not be assigned to an investor’s industry and were thus dropped from the data.

The organizations data was mapped to the full dataset based on the investor/organization names. In some instances, deals had either no investor names filled in or investors were listed as “not reported” or “private” and could thus not be mapped to any organization. These deals had to be dropped, as no investment source country could be identified. This led to an additional elimination in the number of entries in the raw data (all countries, not only OECD) of 10% for equity and 78% for debt investments. We observed no skew in the distribution of deals with missing vs. known investors for technologies, across years or for project countries.

Equity investors were assigned the equity share of a deal and debt investors the debt share, according to the debt shares in the data or the debt shares imputed (see below). For the cases where several investors within the same asset class were involved in a deal, the respective equity or debt values were assigned to the individual participants in equal shares, an assumption that has been applied in previous research working with BNEF project data.^52^^,^^58^

The resulting dataset covered 42,420 deals (45% of the total BNEF asset dataset). We consider this dataset the “raw dataset” (see below Table), which we complemented with additional information based on imputation as described in the next section.

**Table:** Final dataset overview

Dataset	Number of deals	Number of entries	Investment value in US $B
BNEF data (incl. out of scope)	94,566	94,618	N/A
Raw data before imputation	42,420	61,500	N/A
Fully imputed dataset	42,291	61,340	1,944
Share with imputed date	1.1%	1.1%	3.2%
Share with imputed value	78.2%	68.5%	49.8%
Share with imputed debt share	85.4%	70.1%	50.8%
Equity-only dataset (only data with investor type assignment)	37,972	41,691	991
Share with imputed date	1.2%	1.4%	4.6%
Share with imputed value	77.9%	75.9%	61.9%
Share with imputed debt share	86.0%	84.2%	79.5%

#### Imputation of missing data

Despite BNEF being one of the most exhaustive datasets available on RE real asset investments, there are several instances of missing data even within known and reported deals. This type of missing information is comparable to the issue of item nonresponse (as opposed to unit nonresponse, where the entire observation is missing) often encountered and handled in survey-based research methods. While it is possible to simply drop any incomplete observations to do a “complete case analysis”, this may be problematic as it could lead to relevant information being ignored or biases being introduced.^59^^,^^60^^,^^61^ Therefore, a common approach to this problem is the imputation of missing data points.^59^^,^^60^^,^^61^ While it is not possible to infer the investors involved in a deal if no information/name was given (as mentioned above), we did impute missing values based on known values in the data for three types of variables: date of close, investment amounts, and debt values. The imputation models used for the different types of data were the following.

##### Date of close

For 1.1% of entries in the raw dataset, the date of close, which we use to assign the deals to a specific year in our analysis, was missing. For these cases, however, a variable called “date of announcement”, meaning the first date a deal was publicly announced, was available. Using that variable as a basis, we applied a group mean imputation method.^62^ Specifically, we calculated the number of days that typically passed between the announcement and close of the deals (lag days) in cases where both dates were known. We then calculated the average of such “lag days” per technology. Finally, the average lag days for the relevant technology were added to the dates of announcement to impute the missing dates of closing. We chose the group mean imputation method for the date of close variable as it is based on the very simplistic and widely used “mean imputation method” while still allowing us to capture variance for different technologies. The suitability of a “simple” mean versus a group mean imputation method typically depends on the specific dataset.^63^ As we both expected a variance of such lag days between different technologies and also found this to be the case in the data, we decided to add this dimension. While other variables besides technology (e.g. year, country) could have been added to create our groups, this would have added additional complexity with no impact on the end results in our analysis and was therefore not implemented.

##### Investment amount

The total amount of the transaction is not reported for 78% of the deals used in the final data. To impute missing values for this variable, we tested several different imputation models widely used across different types of data and research fields to identify the best fit for our data: linear regression, k-nearest neighbors (kNN), multiple imputation by chained equations (MICE – both predictive mean matching (PMM) and linear regression), and two models based on random forests (missForest and random forest regression).^64^^,^^65^^,^^66^^,^^67^^,^^68^ For this purpose, we used the portion of the data where the investment amount was known. The imputation models were then trained on 80% of that data and subsequently used to impute the values for the test data, i.e. the remaining 20% of the known data. The resulting investment amounts were then contrasted with the true/known investments using root mean squared errors (RMSE), one of the most widely used performance measures for comparing imputation methods.^69^ We chose the random forest regression model, as both random forest models outperformed the other model types (for details see supplemental information: Figure S6; Table S2). Imputation methods based on random forests have become broadly used when dealing with missing data as they (1) work for mixed types of data,^70^ (2) do not require distributional assumptions and can accommodate nonlinear relations/interactions,^68^ and (3) can scale to high dimensions without the risk of overfitting.^71^ Further, the random forest regression model we chose is based on the original random forest proximity algorithm proposed by Breiman,^72^ which produces a large number of trees and then picks the best option to form its prediction. This in turn allowed us to get an understanding of the variability (i.e., standard deviation) in the predictions of the model, which we used for a robustness check of the imputation results (see below for more detail). The model was implemented based on the dataset of 22,963 deals (this includes non-OECD) where investment values were known using the following variables: capacity in MW, country, financing type (e.g., balance sheet), year, transaction type (e.g., new build), technology, deal type (e.g., portfolio), deal status, and a flag for deals that covered a pilot project.

##### Debt and equity

Many deals are financed both by equity and debt, with different investors and lenders per asset class. To assign each investor/lender (and their country) their share of the deal, it is thus required to distinguish the amounts of debt and equity per deal. However, these values are only available for a small share of the deals (4.6% for equity and 13.6% for debt in the final data). Where no equity or debt amounts were available, we therefore had to impute a debt share based on transactions where such a share was known. We again tested several types of models for this imputation, and the random forest models again outperformed the other types of models (for details see supplemental information: Figure S7; Table S3). As can be observed in Figure S7, there is a substantial bulking of debt shares at 70% and 80% in the data, which does not seem to be replicable through imputations. The independent variables available in the data that can be used for the imputation accordingly do not carry the same explanatory power to infer debt shares as they did for the investment amount imputation. Even the best-performing model was only able to achieve an *R*^2^ of 25% (as opposed to 65% for the investment amount imputation above). We therefore added an additional sense check by triangulating the imputed debt shares with data from the AURES II project.^73^ As part of that project, financing data was collected (including debt shares) via 93 semi-structured interviews. This data covers 22 OECD countries and provides 209 empirically elicited technology-specific (solar PV and wind) values for debt shares from 2015–2020 (96% of observations from 2018/2019). For the countries and technologies available in this dataset, we contrasted this additional data with imputed debt shares by country and technology. In this comparison, the imputed debt shares were exactly within the ranges reported there for 57.1% of the deals, and the remainder of the estimates were not more than 35 percentage points outside this range – see Table S4 for further detail. The model was implemented based on the dataset of 6,162 deals (this includes non-OECD) where debt and equity values were known using the following variables: total investment amount, capacity in MW, country, financing type (e.g., balance sheet), year, transaction type (e.g., new build), technology, deal type (e.g., portfolio), deal status, and a flag for deals that covered a pilot project.

#### Modelling uncertainty

Given the share of imputations required (45% of the projects in terms of MW – see Figure S8 for a breakdown of imputed shares per technology), we implemented two robustness checks to test if and how the modelling uncertainty impacted the analysis results – both in terms of the choice of the imputation model as well as the variability within the chosen model. We did this for the investment value and debt share imputations, as these directly impact the final investment amounts assigned to different countries and investors and are accompanied by the largest model uncertainty (vs. date of close, which has a very low error potential). First, we conducted a complete case analysis, meaning that we ran all our analyses with the unimputed data only (i.e., we only included deals where deal value and debt shares were known). The results can be found in the supplemental information (Figures S15–S20). Key insights derived in our paper (e.g., key inflow and outflow countries, increased correlation of RE new outflows and FDI over time for two out of the four technologies, internationalization tendencies of technologies, and outflow share patterns by different investor types) also hold when analysing unimputed data only. Accordingly, the chosen imputation models do not seem to alter the data substantially.

In a second robustness check, we used Monte-Carlo simulations to perform error propagation to identify how the uncertainty introduced from the two imputed values impacted the output of our analyses.^74^^,^^75^ The random forest model chosen allowed us to extract both the mean (i.e., the estimate for the variable) and standard deviation of the 500 trees generated in the imputation process. Based on these, we ran Monte-Carlo simulations (1,000 runs) calculating the absolute net investment flow per country for all technologies and over the entire timeframe using both the distributions for the investment amount as well as the debt share as inputs of uncertainty. We used truncated normal distributions (Equation 1) with the constraints that investment values cannot go below 0 (i.e. a=0) and debt shares have to remain within the range of 0 to 1 (i.e. a=0 and b=1).

(Equation 1) $$f_{T}\left(x|\;\mu ,\sigma^{2}\right)=\frac{f\left(x|\mu ,\sigma^{2}\right)}{F\left(b\right)-F\left(a\right)}\;\text{for}\;a\leq x\leq b$$

The value and debt-share estimates from our imputation models were used as means (μ) and the respective standard deviations as σ in the distributions described above respectively. Using the output of the Monte-Carlo runs, we were able to construct 95% confidence interval ranges (two standard deviations) around the net investment flows per country. See the supplemental information for Figures 1B and 2A with such uncertainty ranges (Figures S21 and S22). As can be observed, the resulting error bars are relatively small and do not impact our findings.

#### Final dataset

Our final dataset (see Table in data processing section) covers 42,291 deals (37,972 for equity investments only – some deals were excluded as investor type could not be identified given that no industry information was available) and US$ 1,944B investment value (US$ 991B for equity investment only). The key variables we used in our analyses were investor/source country, project/destination country, investor type, investment class (debt/equity), investment in US$ (inflation adjusted with 2020 base year), and year of investment.

### Quantification and statistical analysis

• Imputation method testing: To select the appropriate model for imputation of missing data, the performance of 6 models both for the investment amounts as well as the debt shares were tested. Their respective performance was evaluated by calculating the root mean squared error (RMSE)^69^ between imputed and known values. The RMSE is a comparative measure, and a lower number indicates a better fit of the model (see Tables S2 and S3).
• Monte-Carlo simulations: To test the potential uncertainty introduced by the imputations we ran Monte-Carlo simulations based on the distributions of investment and debt share amounts. The resulting output allowed us to construct confidence intervals (95%) around the mean net investment flows per country (that is used throughout the study). Figures S21 and S22 show the mean +/- 2 SD ranges (derived from the simulations) for key figures. We can observe that the potential uncertainty introduced does not alter key findings (based on visual insights and patterns) we derive from the figures throughout the study.

## Supplemental information

Document S1. Figures S1–S22 and Tables S1–S4

Table S5. Supplementary Excel file containing mapping logic used to assign investor type classes to BICS industries

Table S6. Supplementary Excel file containing the mapping logic used to assign investor type classes to sub-activities
