# Supplementary material for: The internationalization of renewable energy finance
Source: iScience. 2025 Apr 6;28(5):112367. doi: 10.1016/j.isci.2025.112367 (PMC12052835; doi:10.1016/j.isci.2025.112367)
Supplement: Document S1. Figures S1–S22 and Tables S1–S4 [file mmc1.pdf]

iScience, Volume *28*

Supplemental information

The internationalization of renewable energy finance

Sara Eberhart, Tobias S. Schmidt, Bjarne Steffen, and Florian Egli

Figures

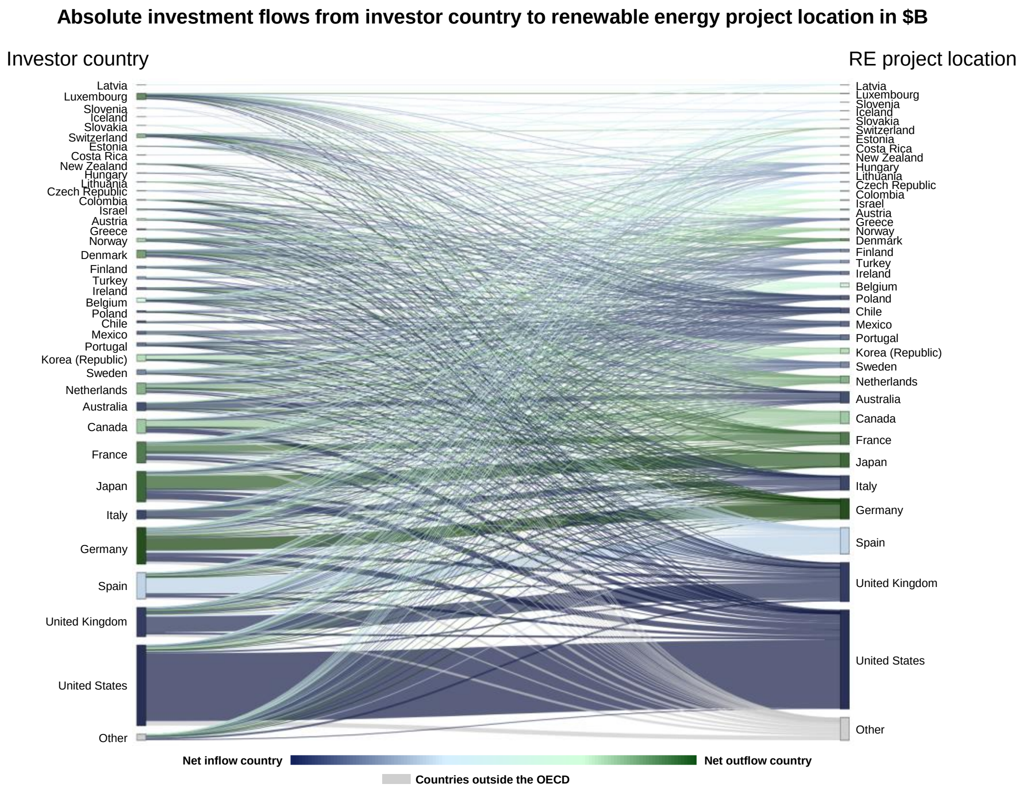

Fig. S1. Annual RE investment in- and outflows per OECD country 2004–2022, Related to Figure 1

RE investment flows in US$B connecting investor and investee countries via investor and project location for all OECD countries. Data is aggregated across all RE technologies considered from 2004 to 2022 and shown in 2020 US$.

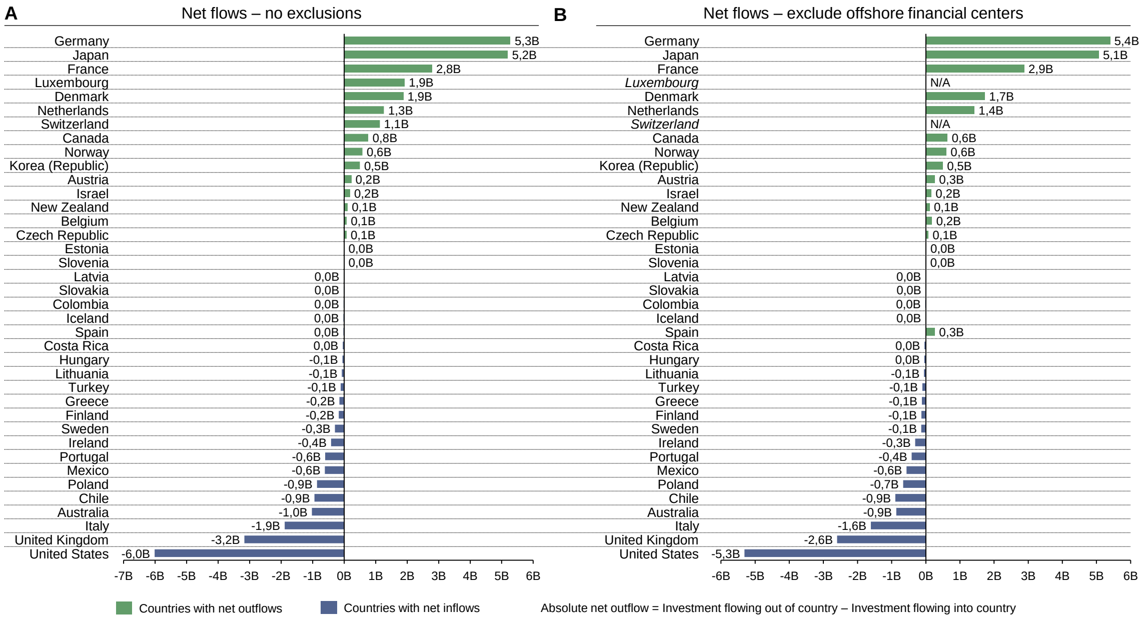

Fig. S2. Annual RE investment net outflows per country 2004–2022 with and without OFC, Related to Figure 1

A Analysis including all countries. **B** Analysis excluding all countries classified as Offshore Financial Centres based on the 2007 definition of the IMF working paper: “Concept of Offshore Financial Centers: In Search of an Operational Definition” (2 observed standard deviations). Data is aggregated across all RE technologies considered from 2004 to 2022 and shown in 2020 US$.

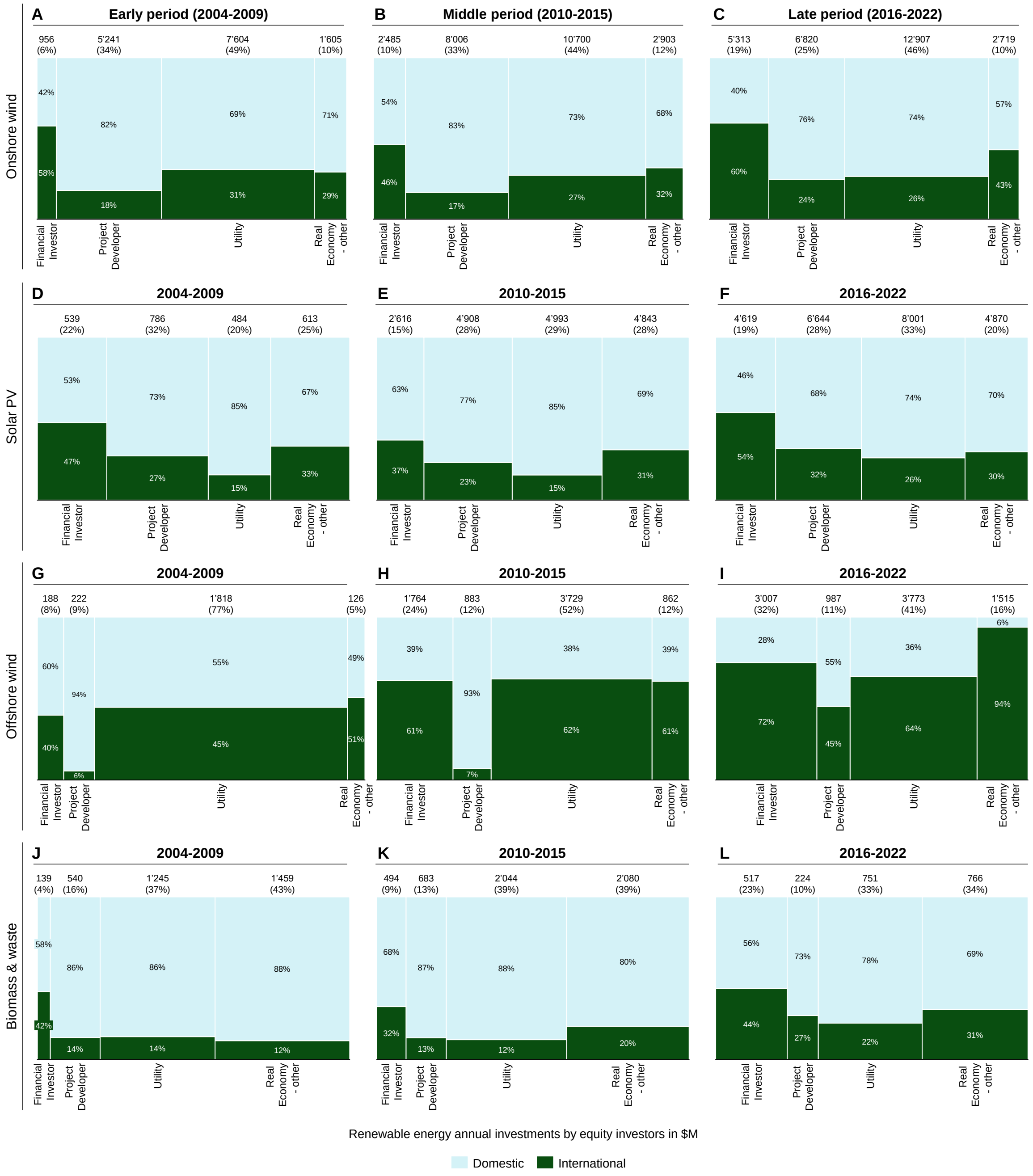

Fig. S3. Domestic and international equity investments by investor types over time, Related to Figure 4

By technologies: **A–C.** onshore wind, **D–F.** solar PV, **G–I.** offshore wind, **J–L.** biomass & waste. The area of each chart reflects the relative distribution of domestic versus international investments and the relative share invested by type of equity investor. The shares of domestic and international investments per investor type are indicated by the percentage numbers within the areas. The absolute annualized investment amounts and the corresponding relative share of investments per investor type and time period is indicated at the top of each column. Absolute investment numbers are in shown 2020 US$.

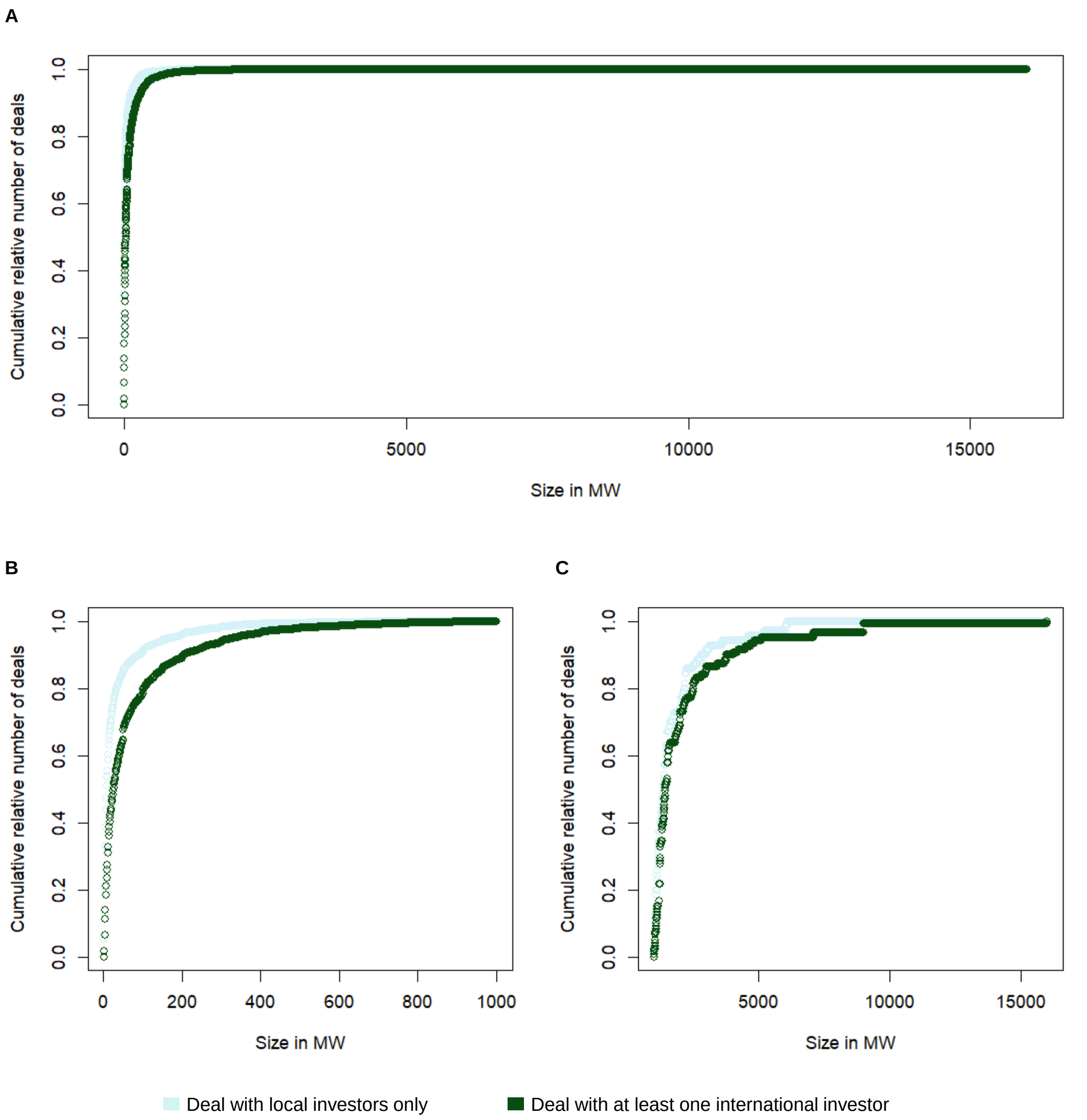

Fig. S4. Cumulative relative number of deals by project size in MW, Related to discussion

A All deals. **B** Deals for projects with 0-1,000 MW. **C** Deals for projects with 1,001-16,000 MW. Cumulative number of deals have been standardized by number of deals by type of deal to make local and international deals comparable. All deals are split up in **B** & **C** to better show differences that are not visible on a larger scale. Data is considered from 2004 to 2022 and includes investments from/to OECD countries

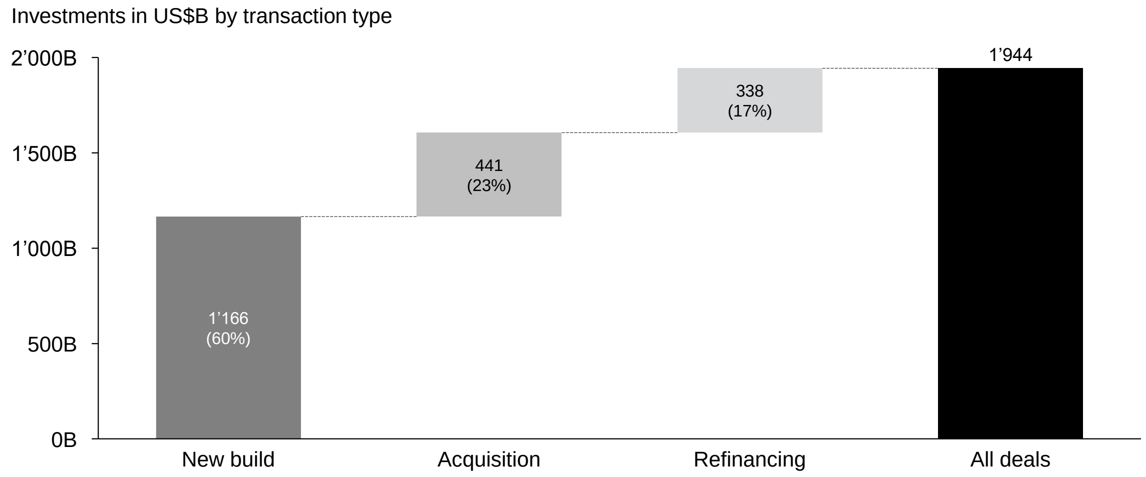

Fig. S5. Final dataset investment shares by transaction type, Related to STAR Methods

Absolute and relative share of investments in final (imputed) dataset by three reported transaction types: New build, acquisition, and refinancing. Data is considered from 2004 to 2022, includes investments from/to OECD countries and absolute investment numbers are shown in 2020 US$.

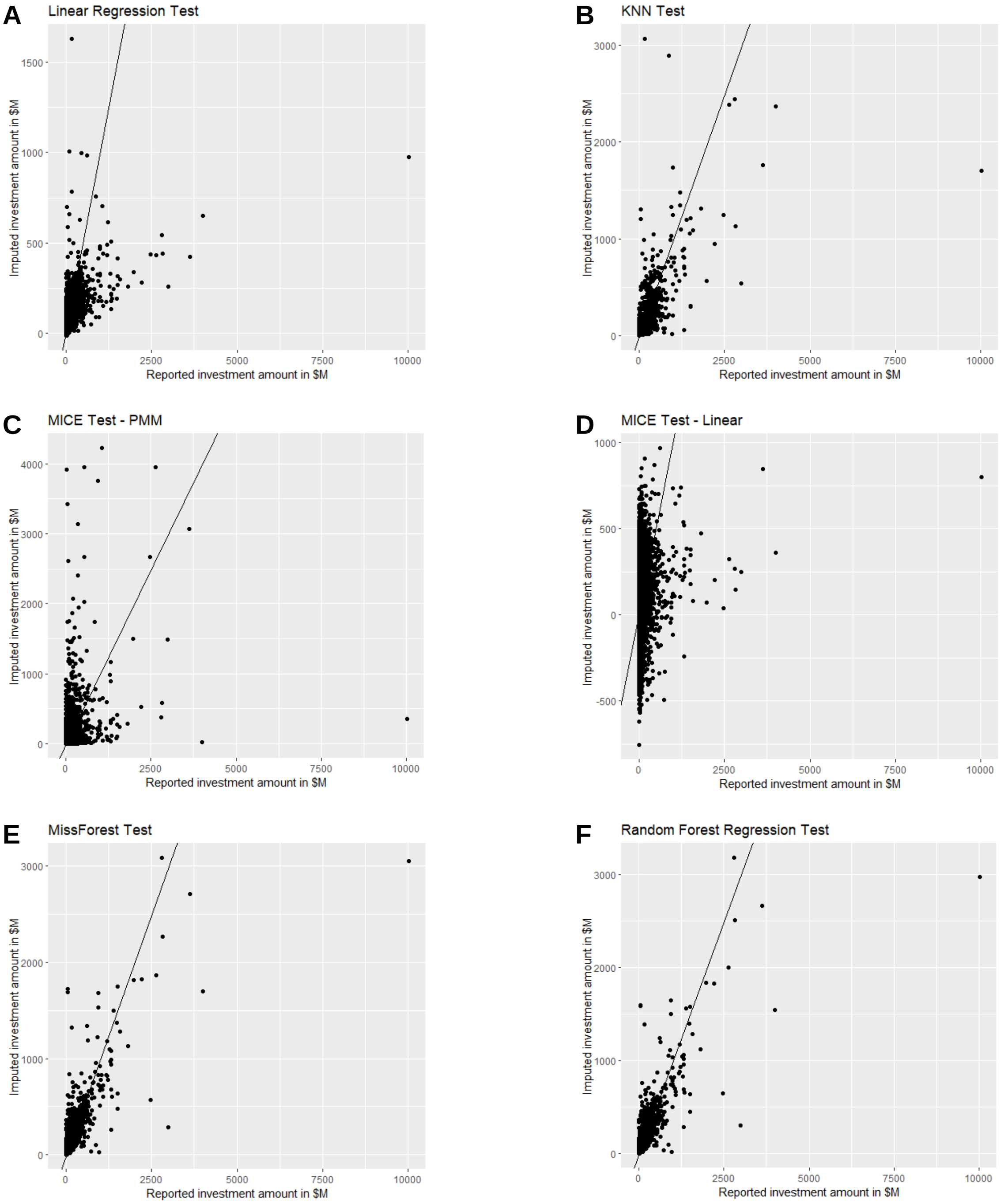

Fig. S6. Imputation model tests: Imputed versus reported investment amount, Related to STAR Methods

For the imputation model selection, six models were run on a test dataset and compared with known values for the investment amounts. The graphs visualize the comparison of imputed and known/reported amounts. For readability purposes a line with slope 1 and intercept 0 is added. Models tested are: **A** linear regression. **B** k-kearest neighbors (kNN) (k = 10). **C** multiple imputation by chained equations with predictive mean matching (PMM) (m = 10). **D** multiple imputation by chained equations with linear regression using bootstrap (m = 10). **E** missForest (R package default settings). **F** random forest regression (R package default settings).

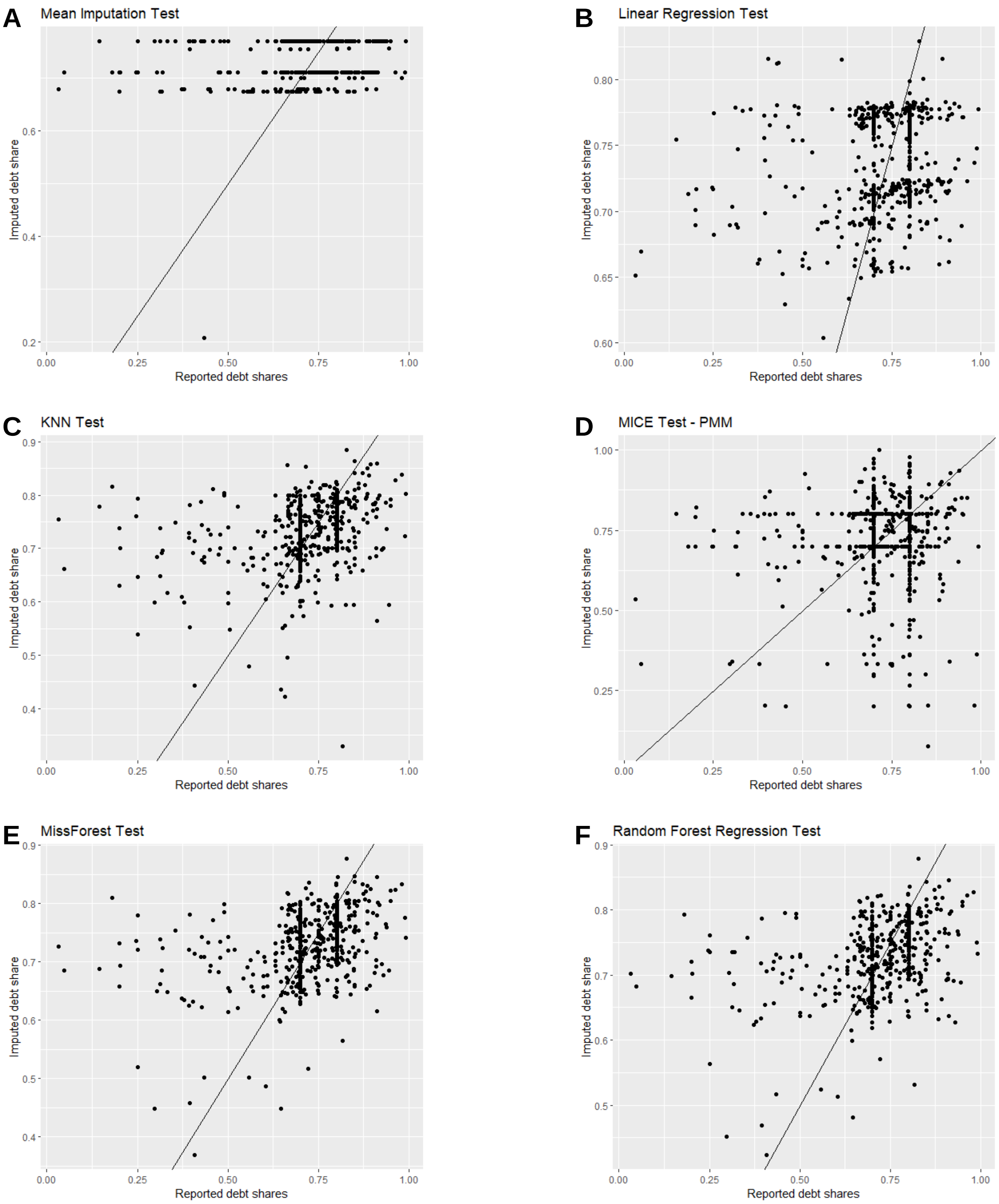

Fig. S7. Imputation model tests: Imputed versus reported debt share, Related to STAR Methods

For the imputation model selection, six models were run on a test dataset and compared with known values for the debt shares. The graphs visualize the comparison of imputed and known/reported amounts. For readability purposes a line with slope 1 and intercept 0 is added. Models tested are **A** mean imputation. **B** linear regression. **C** k-nearest neighbors (kNN) (k = 10). **D** multiple imputation by chained equations with predictive mean matching (PMM) (m = 10). **E** missForest (R package default settings). **F** random forest regression (R package default settings).

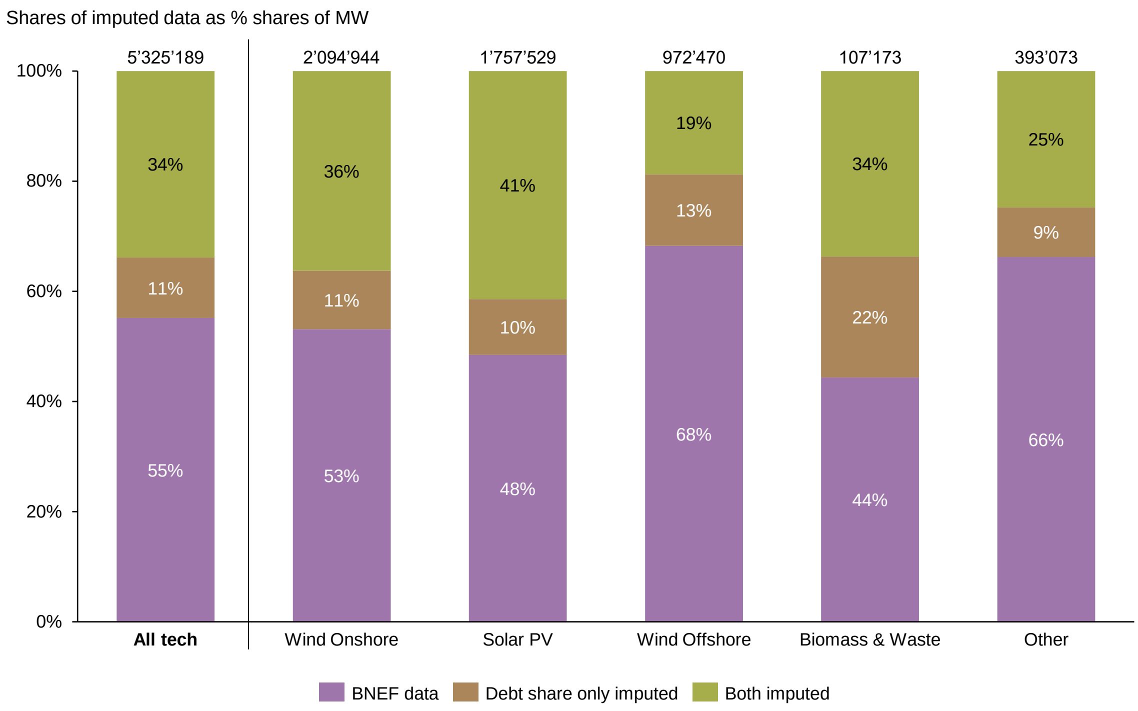

Fig. S8. Shares of imputed data in final dataset in relation to project MW, Related to STAR Methods

To illustrate the representativeness of the data, the share of imputations based on the MW of the financed projects are split into original BNEF data, deals where only debt shares were imputed, and deals where both debt share and investment amounts were imputed. This is done both for the full dataset as well as split by technology. The numbers above the bars refer to the total MWs. The data is aggregated over the entire time period spanning 2004 to 2022.

Figures including "new build" data only

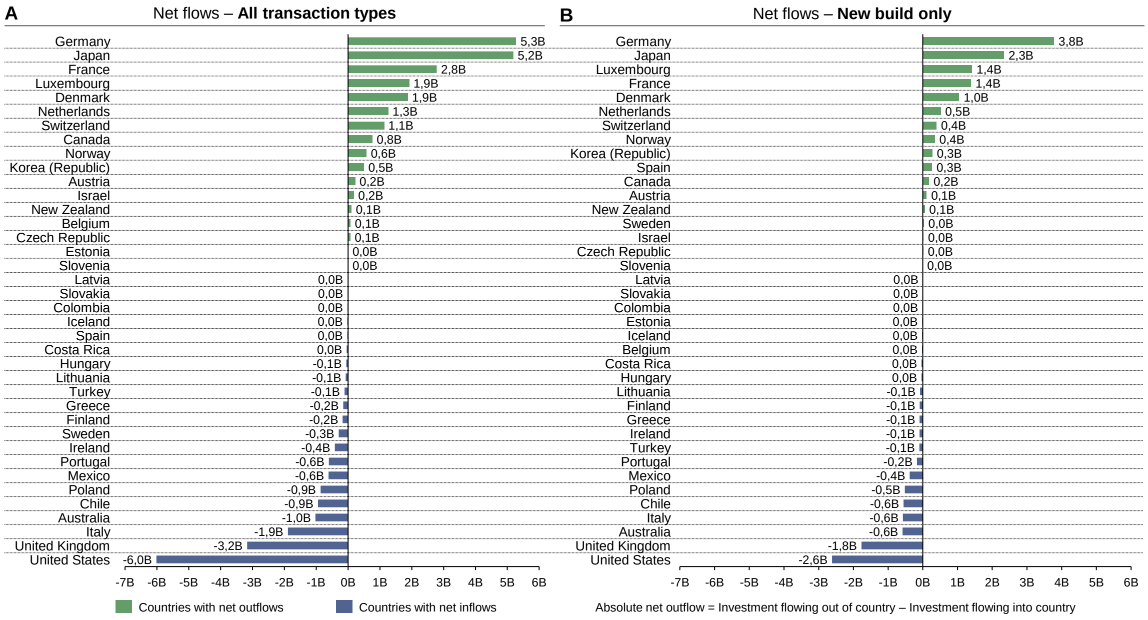

Fig. S9. Annual RE investment net outflows per country 2004–2022, Related to Figure 1

A Analysis including all data. **B** Analysis only including data for "new build" investments. Data is aggregated across all RE technologies considered from 2004 to 2022 and shown in 2020 US$.

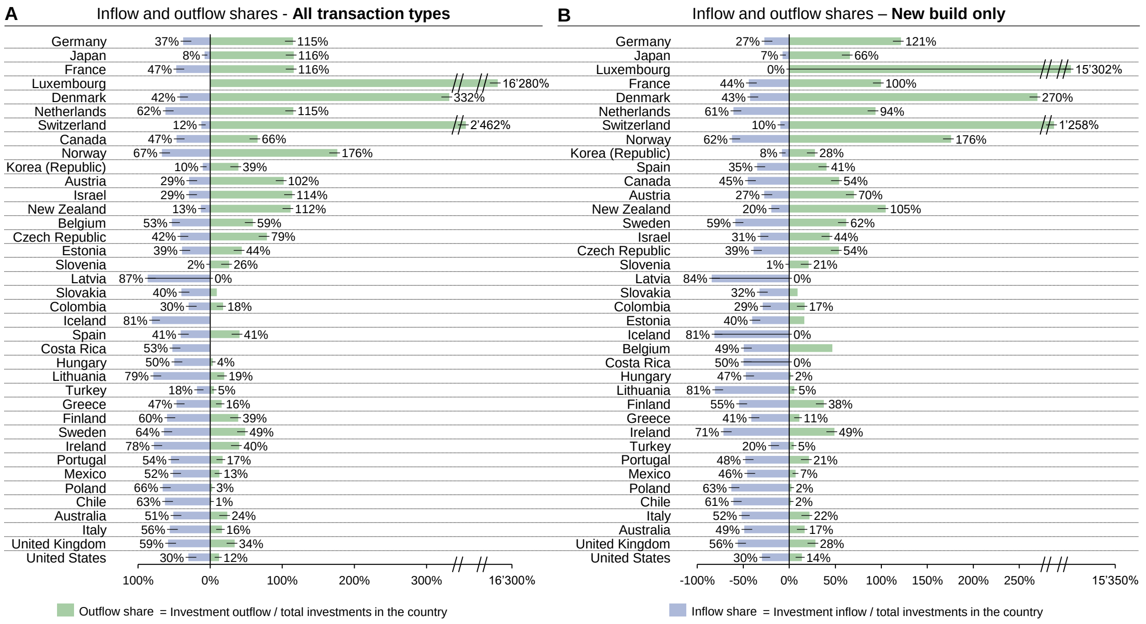

Fig. S10. Annual RE investment inflow and outflow shares per country 2004–2022, Related to Figure 1

Outflow share = investment outflow / total investments in the country. Inflow share = investment inflow / total investments in the country **A** Analysis including all data. **B** Analysis only including data for "new build" investments. Data is aggregated across all RE technologies considered from 2004 to 2022.

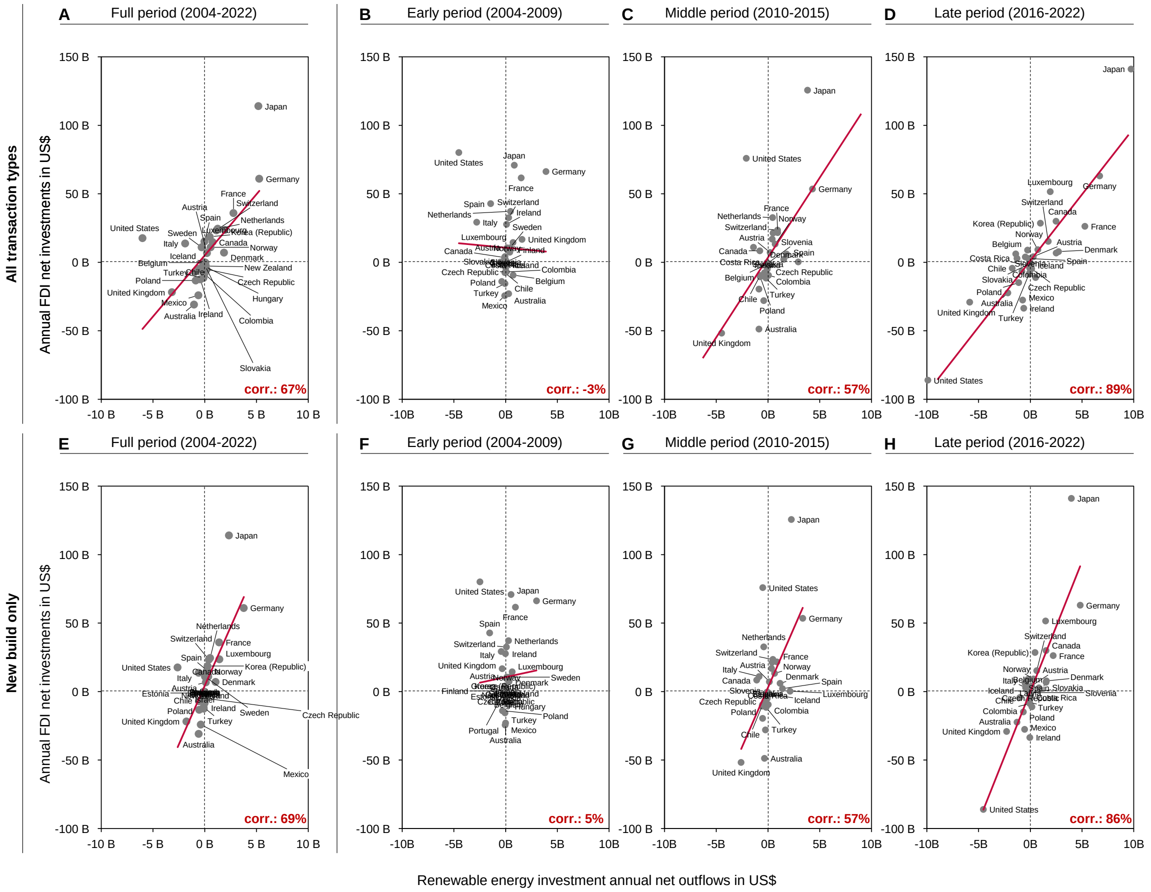

Fig. S11. Annual absolute net investment flows into RE contrasted with foreign direct investments per country, Related to Figure 2

A–D Analysis including all data. **A** Aggregated from 2004 to 2022. **B** Aggregated for the early period (2004–2009). **C** Aggregated for the middle period (2010–2015). **D** Aggregated for the late period after the Paris Agreement (2016–2022). **E–H** Analysis only including data for "new build" investments. **E** Aggregated from 2004 to 2022. **F** Aggregated for the early period (2004–2009). **G** Aggregated for the middle period (2010–2015). **H** Aggregated for the late period after the Paris Agreement (2016–2022). The red line represents the trendline of the observations. "Corr." Indicates the simple correlation between the net RE outflows and FDI Data is aggregated across all RE technologies and shown in 2020 US$.

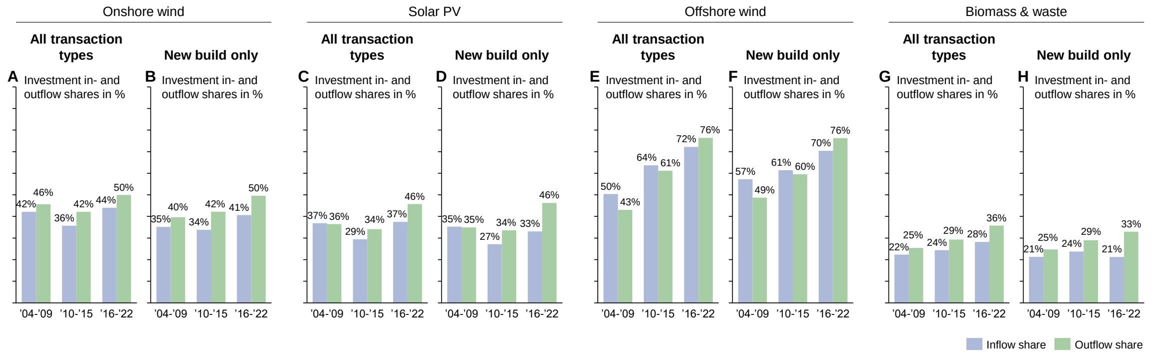

Fig. S12. Investment inflow and outflow shares aggregated across all countries, Related to Figure 3

Analysis including all data for **A** onshore wind, **C** solar PV, **E** offshore wind, **G** biomass & waste. Analysis only including data for "new build" investments for **B** onshore wind, **D** solar PV, **F** offshore wind, **H** biomass & waste.

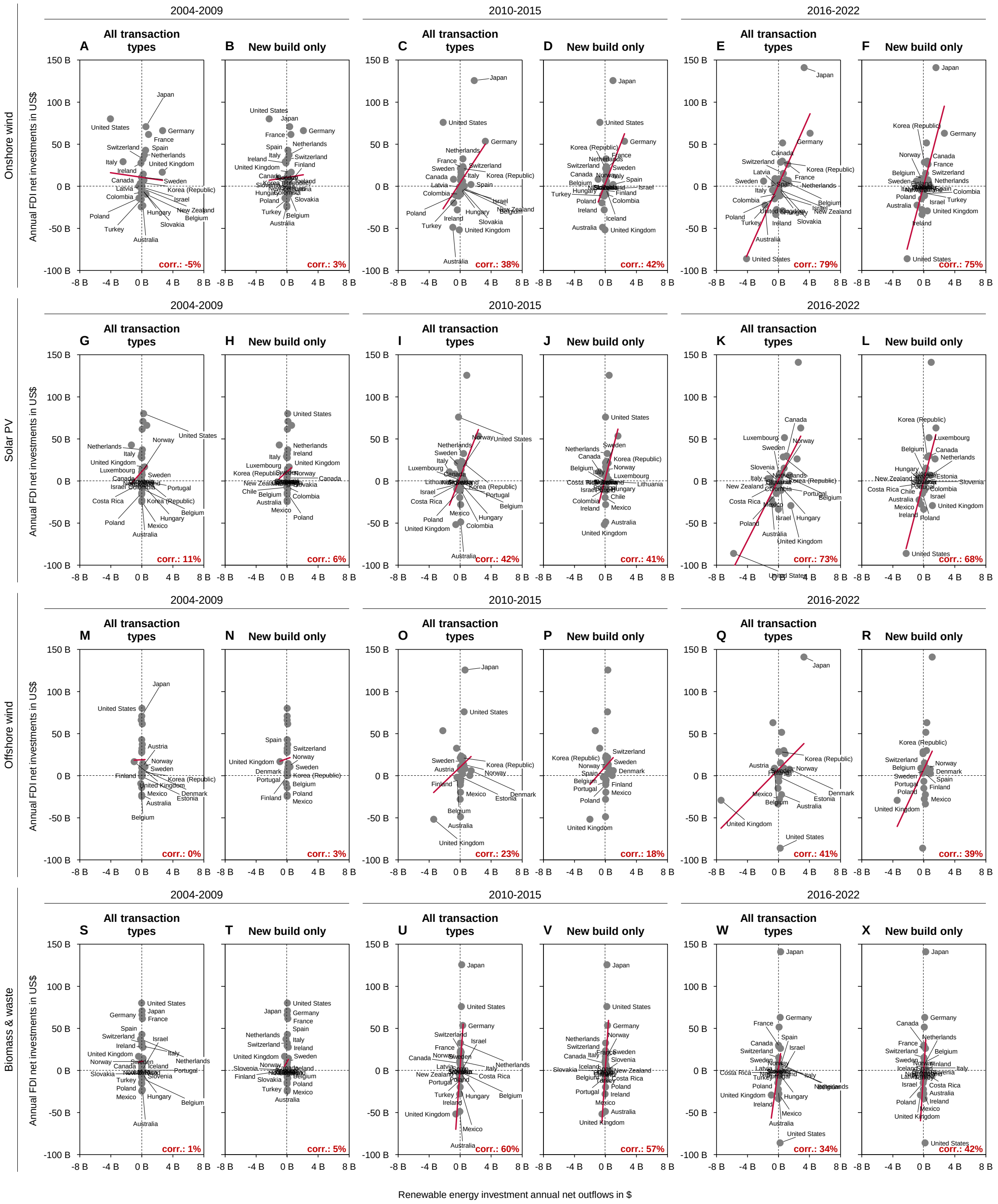

Fig. S13. Annual absolute net investment flows into RE contrasted with foreign direct investments per country over three time periods, Related to Figure 3

Analysis including all data for **A**/**C**/**E** onshore wind, **G**/**I**/**K** solar PV, **M**/**O**/**Q** offshore wind, **S**/**U**/**W** biomass & waste. Analysis only including data for "new build" investments for **B**/**D**/**F** onshore wind, **H**/**J**/**L** solar PV, **N**/**P**/**R** offshore wind, **T**/**V**/**X** biomass & waste. The red line represents the trendline of the observations. "Corr." Indicates the simple correlation between the net RE outflows and FDI. Absolute investment numbers are shown in 2020 US$.

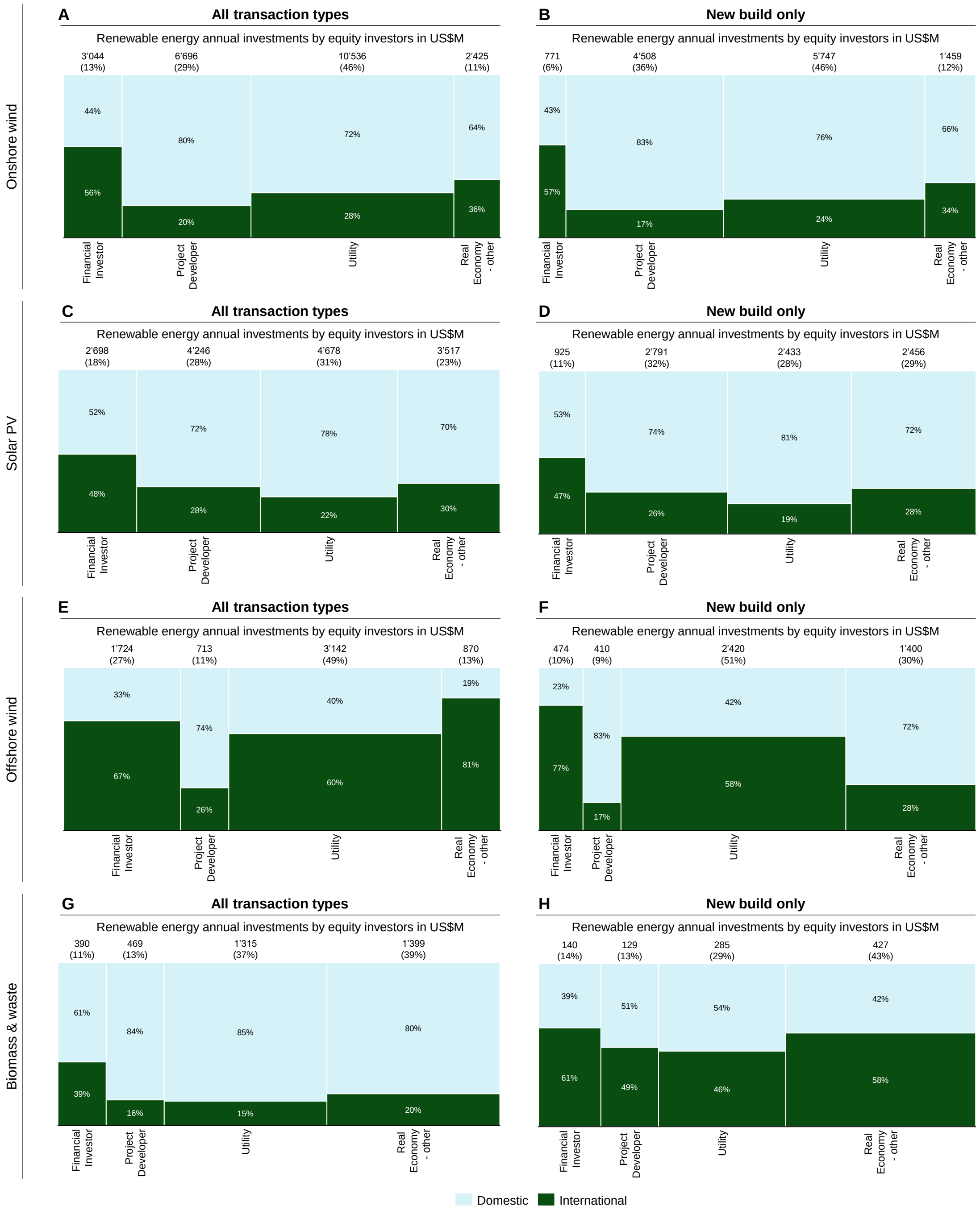

Fig. S14. Domestic and international equity investments by investor types, Related to Figure 4

Analysis including all data for **A** onshore wind, **C** solar PV, **E** offshore wind, **G** biomass & waste. Analysis only including data for "new build" investments for **B** onshore wind, **D** solar PV, **F** offshore wind, **H** biomass & waste. The area of each chart reflects the relative distribution of domestic versus international investments and the relative share invested by type of equity investor. The shares of domestic and international investments per investor type are indicated by the percentage numbers within the areas. The annualized investment amounts and the corresponding relative share of investments per investor type is indicated at the top of each column. Data is considered from 2004 to 2022 and absolute investment numbers are shown in 2020 US$.

Figures without imputations – Complete case analysis

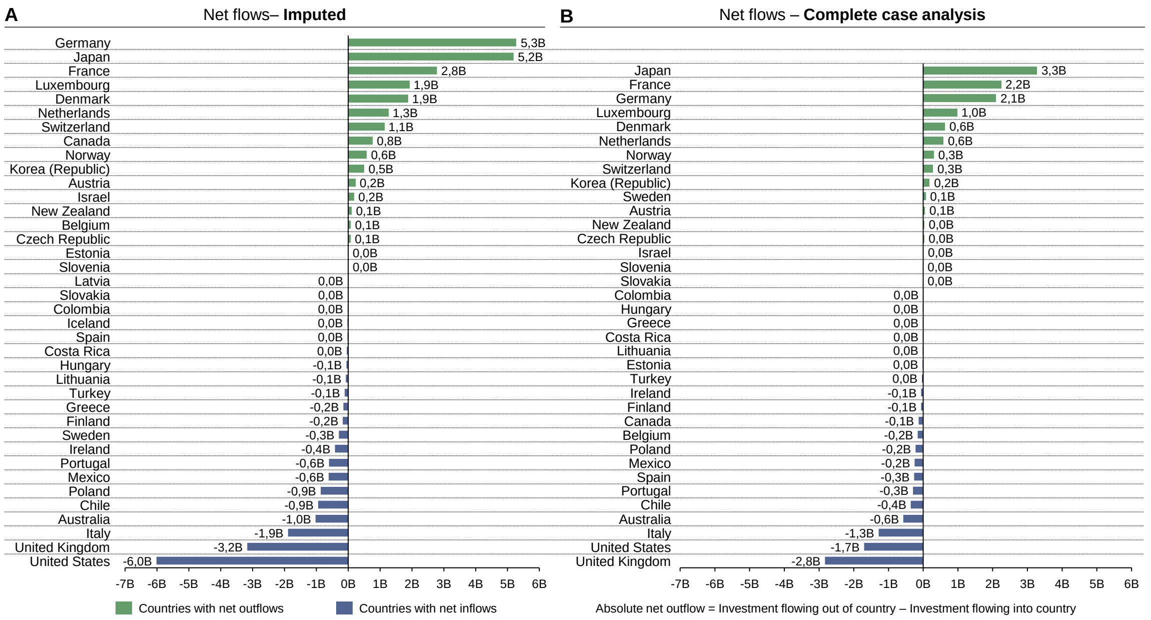

Fig. S15. Annual RE investment net outflows per country 2004–2022, Related to Figure 1

A Analysis including imputed data. **B** Complete case analysis only including known investment amounts and debt shares. Data is aggregated across all RE technologies considered from 2004 to 2022 and shown in 2020 US$.

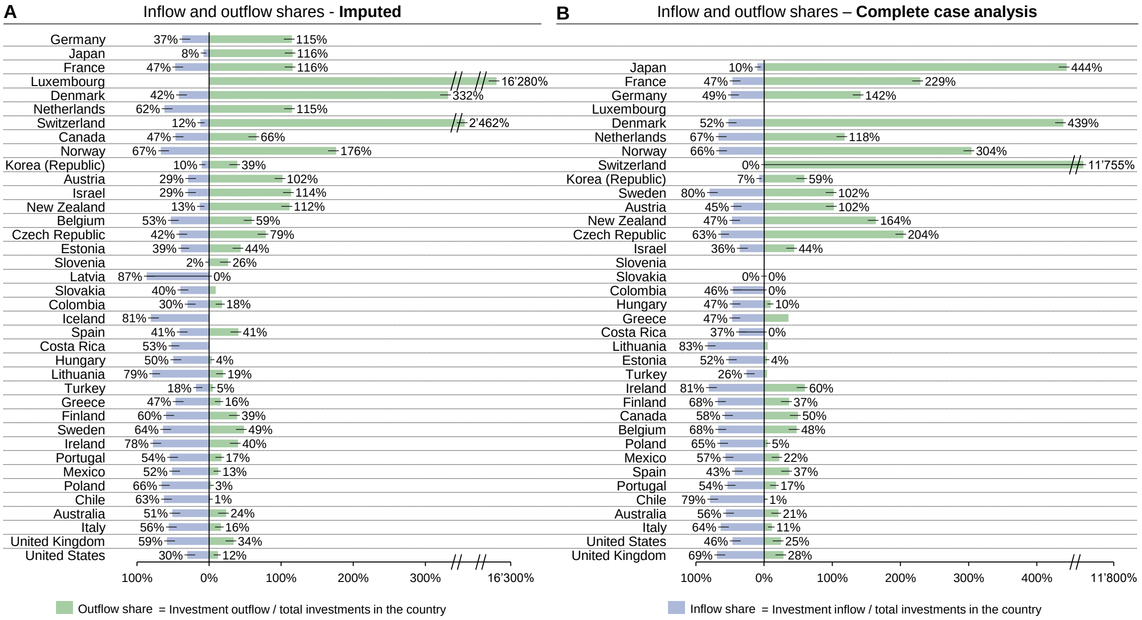

Fig. S16. Annual RE investment inflow and outflow shares per country 2004–2022, Related to Figure 1

Outflow share = investment outflow / total investments in the country. Inflow share = investment inflow / total investments in the country **A** Analysis including imputed data. **B** Complete case. Data is aggregated across all RE technologies considered from 2004 to 2022.

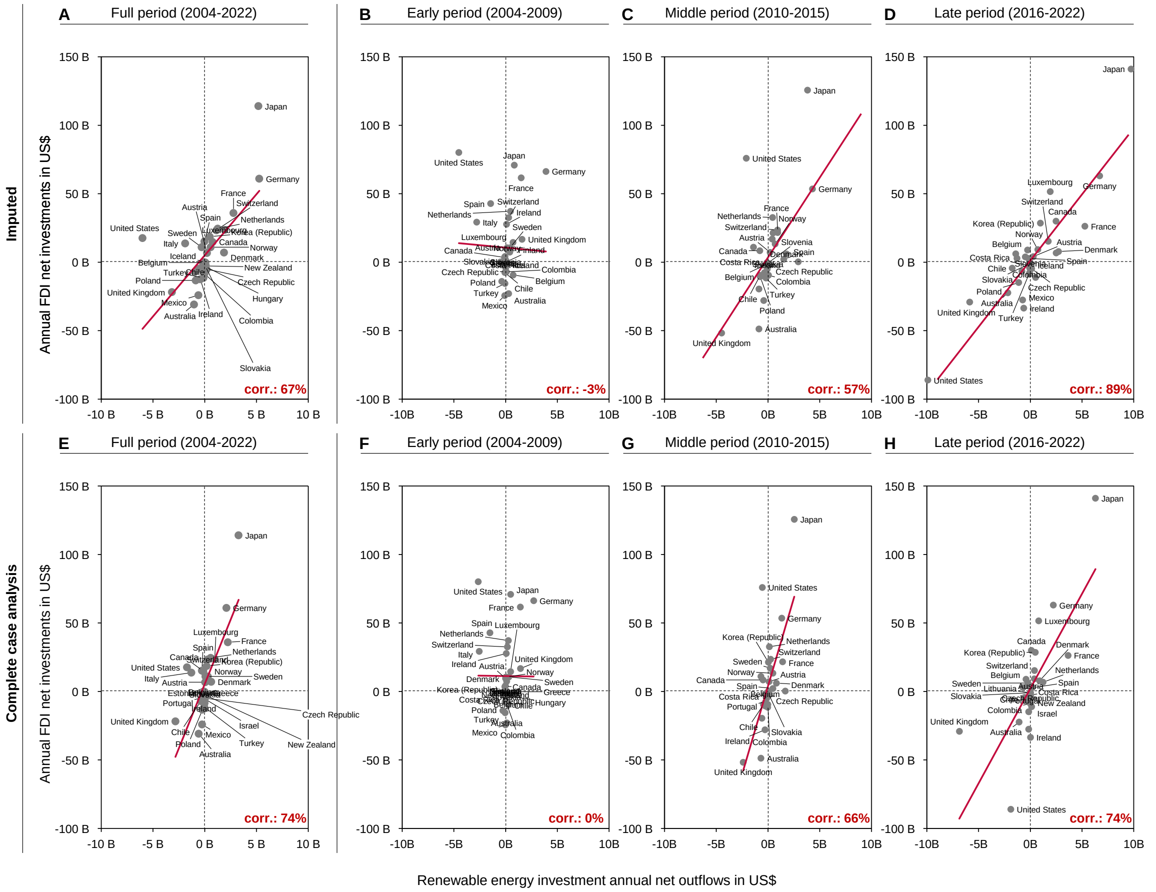

Fig. S17. Annual absolute net investment flows into RE contrasted with foreign direct investments per country, Related to Figure 2

A–D Analysis including imputed data. **A** Aggregated from 2004 to 2022. **B** Aggregated for the early period (2004–2009). **C** Aggregated for the middle period (2010–2015). **D** Aggregated for the late period after the Paris Agreement (2016–2022). **E–H** Complete case analysis only including known investment amounts and debt shares. **E** Aggregated from 2004 to 2022. **F** Aggregated for the early period (2004–2009). **G** Aggregated for the middle period (2010–2015). **H** Aggregated for the late period after the Paris Agreement (2016–2022). The red line represents the trendline of the observations. “Corr.” Indicates the simple correlation between the net RE outflows and FDI Data is aggregated across all RE technologies and shown in 2020 US$.

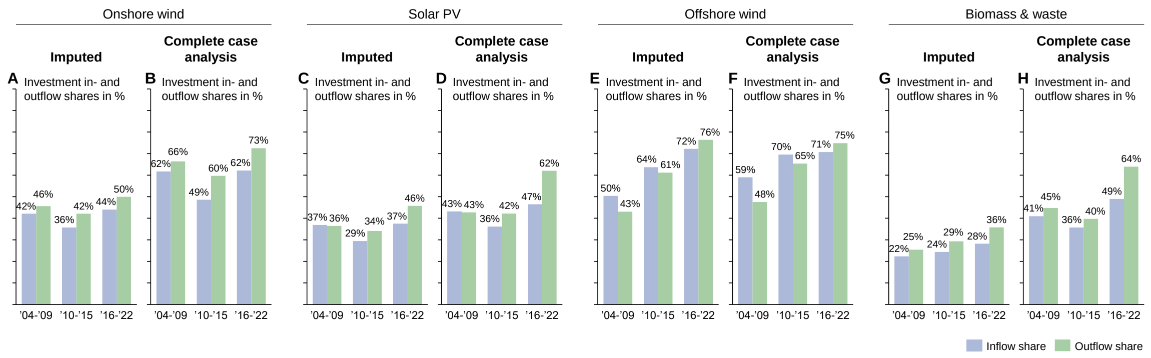

Fig. S18. Investment inflow and outflow shares aggregated across all countries, Related to Figure 3

Analysis including imputed data for **A** onshore wind, **C** solar PV, **E** offshore wind, **G** biomass & waste. Complete case analysis only including known investment amounts and debt shares for **B** onshore wind, **D** solar PV, **F** offshore wind, **H** biomass & waste.

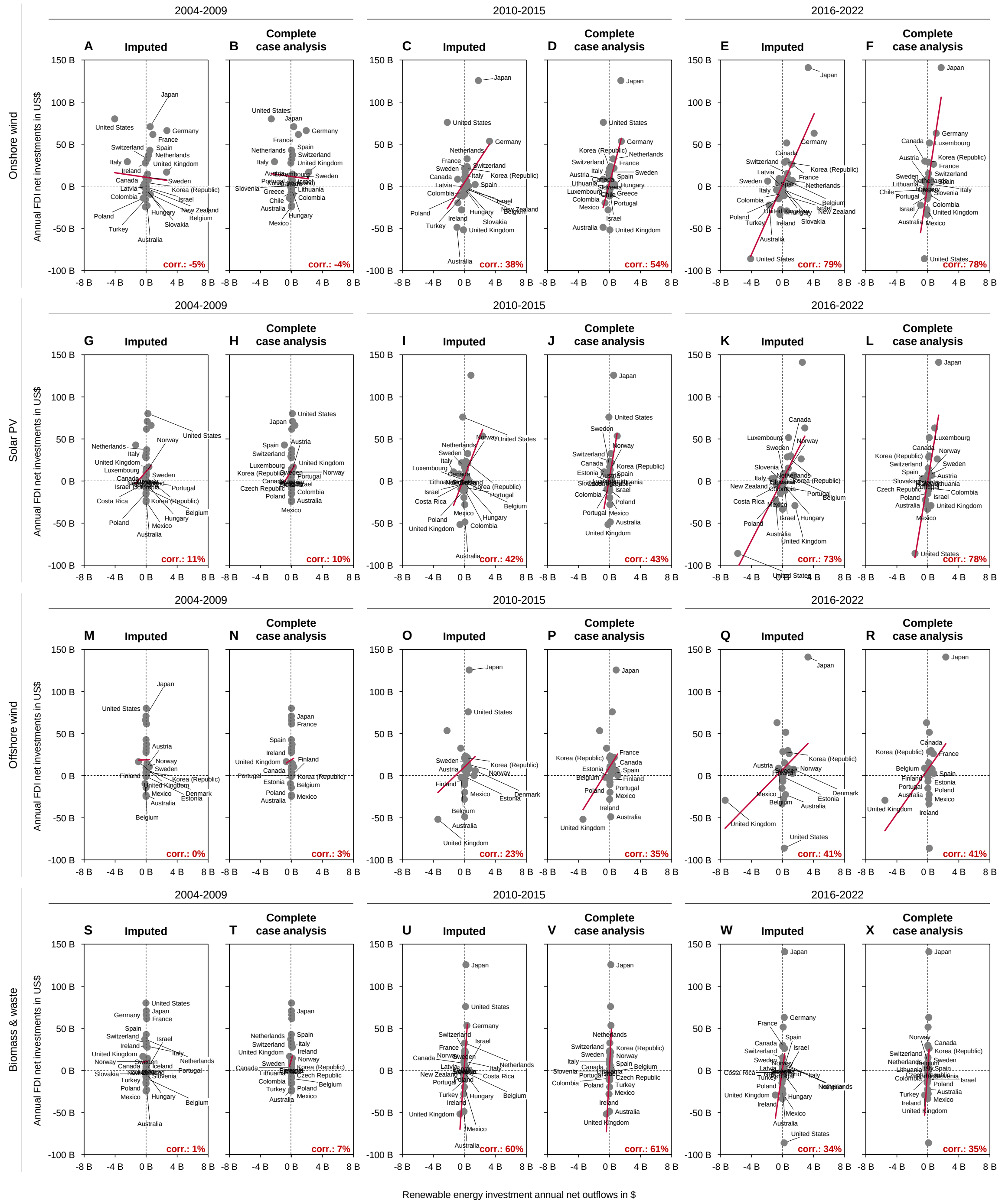

Fig. S19. Annual absolute net investment flows into RE contrasted with foreign direct investments per country over three time periods, Related to Figure 3

Analysis including imputed data for **A**/**C**/**E** onshore wind, **G**/**I**/**K** solar PV, **M**/**O**/**Q** offshore wind, **S**/**U**/**W** biomass & waste. Complete case analysis only including known investment amounts and debt shares for **B**/**D**/**F** onshore wind, **H**/**J**/**L** solar PV, **N**/**P**/**R** offshore wind, **T**/**V**/**X** biomass & waste. The red line represents the trendline of the observations. "Corr." Indicates the simple correlation between the net RE outflows and FDI. Absolute investment numbers are shown in 2020 US$.

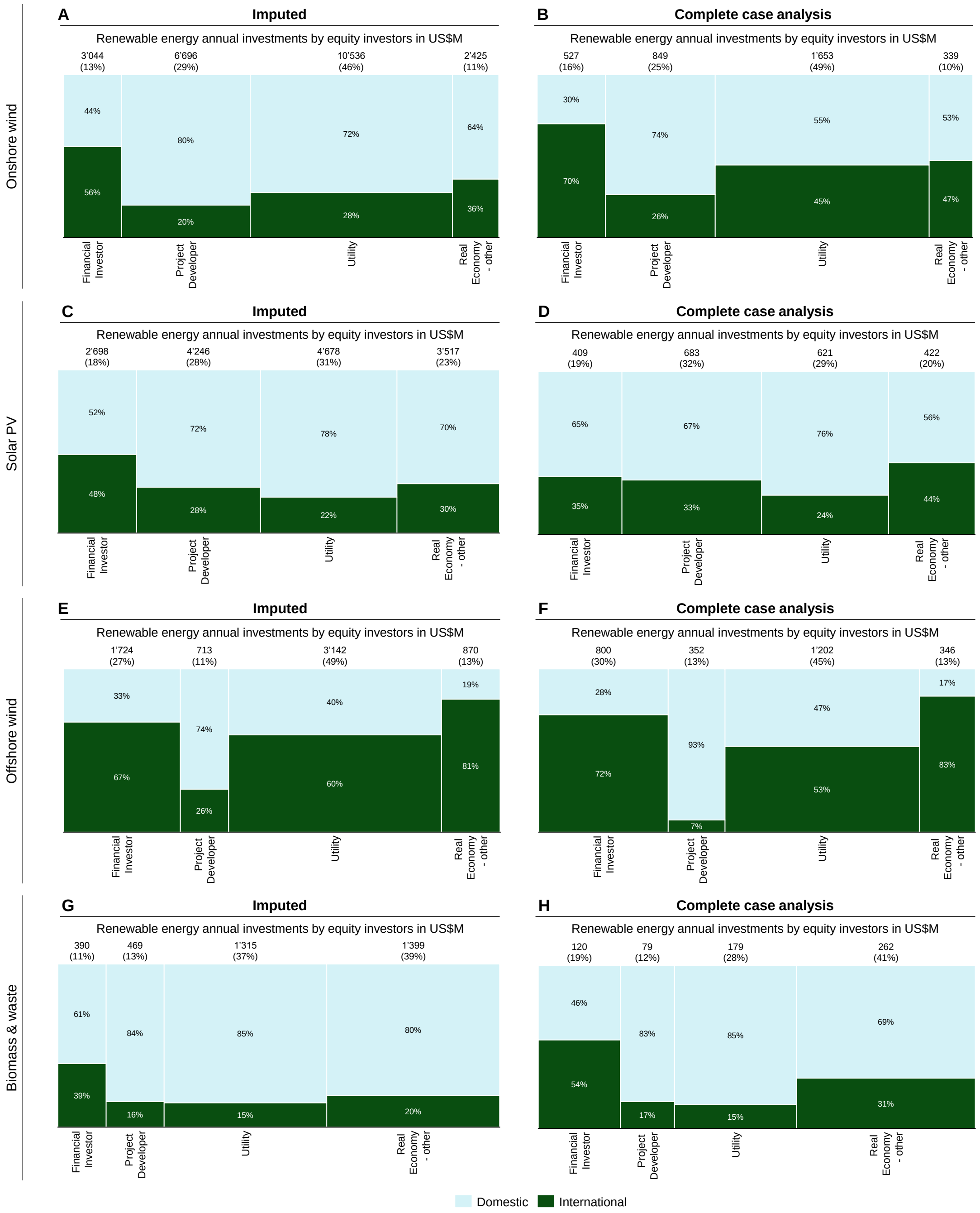

Fig. S20. Domestic and international equity investments by investor types, Related to Figure 4

Analysis including imputed data for **A** onshore wind, **C** solar PV, **E** offshore wind, **G** biomass & waste. Complete case analysis only including known investment amounts and debt shares for **B** onshore wind, **D** solar PV, **F** offshore wind, **H** biomass & waste. The area of each chart reflects the relative distribution of domestic versus international investments and the relative share invested by type of equity investor. The shares of domestic and international investments per investor type are indicated by the percentage numbers within the areas. The annualized investment amounts and the corresponding relative share of investments per investor type is indicated at the top of each column. Data is considered from 2004 to 2022 and absolute investment numbers are shown in 2020 US$.

Figures with ranges

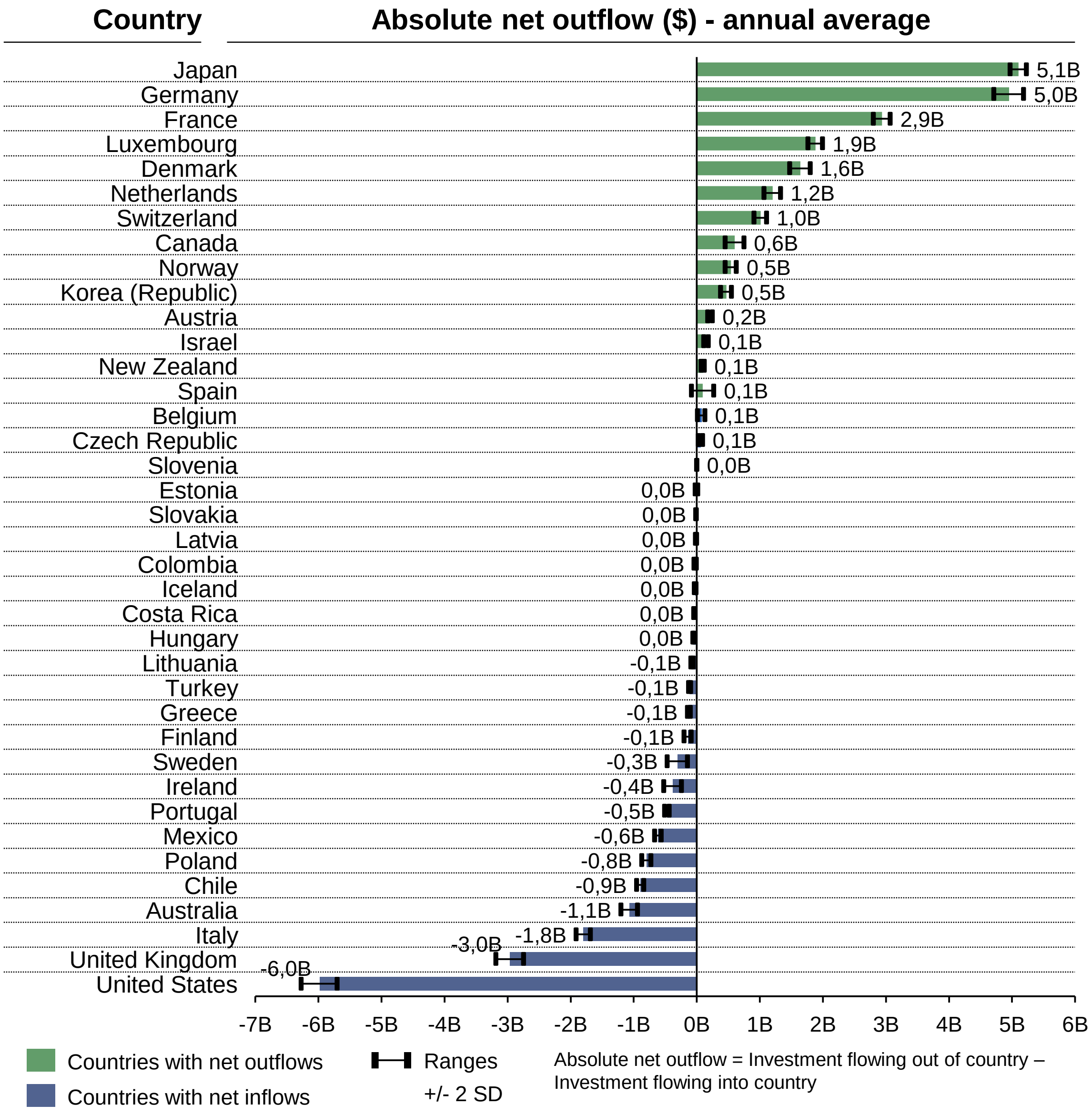

Fig. S21. Absolute net investment flows into renewable energy with model uncertainty ranges, Related to Figure 1

The chart reflects the net flows as an average per year over the entire time period and indicates the model uncertainty with ranges referencing the 95% confidence interval, i.e., data are represented as mean +/- 2 SD. Data is aggregated across all RE technologies considered (onshore and offshore wind, solar photovoltaics (PV), solar thermal, biomass and waste, small-scale hydroelectric, and marine energy) from 2004 to 2022 and in 2020 US$.

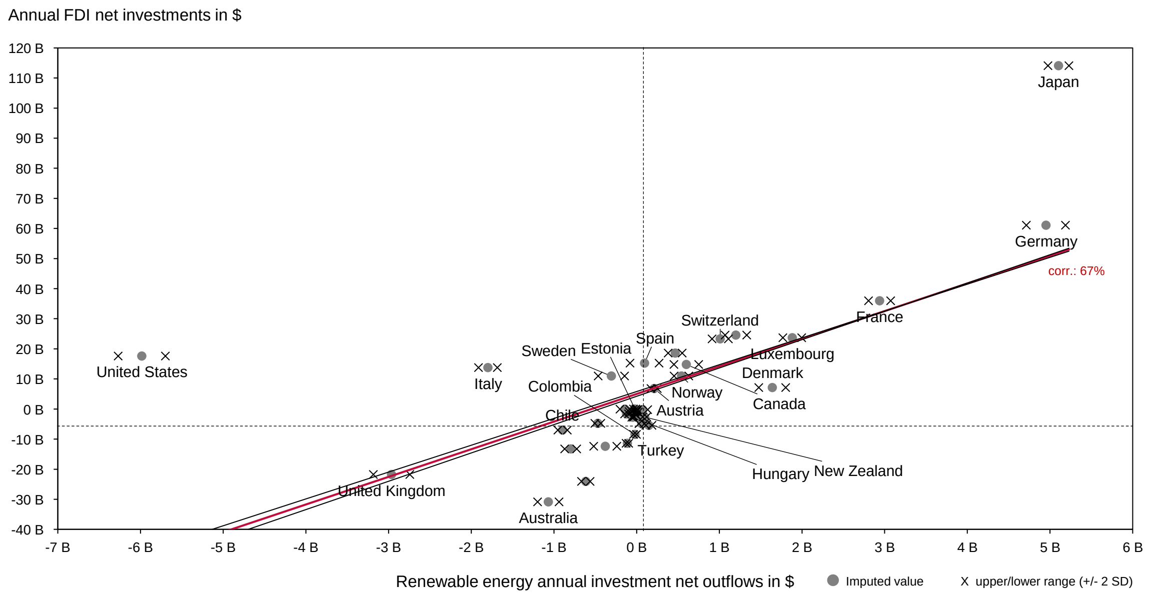

Fig. S22. Absolute net investment flows into renewable energy contrasted with foreign direct investments per country, Related to Figure 2

Each dot depicts the country's net FDI investments in contrast to the absolute net renewable energy investment flows (including trendline and respective correlation coefficient), aggregated over technologies and averaged per year for the entire timeframe 2004-2022. The crosses mark the 95% confidence interval, i.e., data are represented as mean +/- 2 SD, to account for the uncertainty in the investment value estimation process.

Tables

Table S1, Related to Figure 1

15 largest investors based on their RE financing flows into the US. Of these top investors, only Capital Dynamics represents a case where clear circularity of capital can be observed (only contributing 2% of total investment flows into the US). All other investors were banks from distinct (non-Offshore Financial Centre) countries

Nb	Investor	Country	Total US invest in $B	% of total US inflows
**1**	Sumitomo Mitsui Banking Corp	Japan	9.1	5%
**2**	Mizuho Bank Ltd	Japan	7.8	4%
**3**	MUFG Bank Ltd	Japan	6.7	4%
**4**	Banco Santander SA	Spain	6.4	4%
**5**	Hamburg Commercial Bank AG	Germany	6.3	3%
**6**	Norddeutsche Landesbank-Girozentrale	Germany	6.1	3%
**7**	Mitsubishi UFJ Financial Group Inc	Japan	5.7	3%
**8**	Bayerische Landesbank	Germany	4.8	3%
**9**	Cooperatieve Rabobank UA	Netherlands	4.3	2%
**10**	Capital Dynamics AG	Switzerland	4.1	2%
**11**	Societe Generale SA	France	3.3	2%
**12**	UniCredit Bank AG	Germany	3.2	2%
**13**	Natixis SA	France	2.9	2%
**14**	Canadian Imperial Bank of Commerce	Canada	2.6	1%
**15**	Credit Suisse AG	Switzerland	2.3	1%

Table S2, Related to STAR Methods

Model performance for investment amount imputation as measured by root mean squared error (lower errors indicate a better fit).

Imputation model	RMSE
Linear regression	215.7
KNN	176.4
MICE – PMM	280.5
MICE – linear	299.0
MissForest	148.3
Random Forest regression	148.2

Table S3, Related to STAR Methods

Model performance for debt share imputation as measured by root mean squared error (lower errors indicate a better fit).

Imputation model	RMSE
Mean imputation	0.094
Linear regression	0.093
KNN	0.090
MICE – PMM	0.137
MissForest	0.086
Random Forest regression	0.086

Table S4, Related to STAR Methods

Table of debt share triangulation with AURIS II dataset

**Distance to AURIS debt share range (in percentage points)**	Inside	<5%	> 5% < 10%	> 10% < 20%	> 20% < 35%
**Share of deals in raw dataset for which imputed debt shares come to lie in/outside AURES debt share range**	57.1%	5.7%	5.9%	25.4%	5.8%
